# Acinar-specific loss of activating transcription factor 3 restricts KRAS^G12D^ mediated transcriptional changes and PanIN progression

**DOI:** 10.1038/s41420-025-02777-2

**Published:** 2025-11-06

**Authors:** Mickenzie B. Martin, Fatemeh Mousavi, Gavin Goebel, Domenic Di Stasi, Alesia Mano, Andrei Glogov, Liena Zhao, Gianni Di Guglielmo, Parisa Shooshtari, Christopher L. Pin

**Affiliations:** 1 Department of Physiology and Pharmacology, Schulich School of Medicine and Dentistry, Western University, London, ON Canada; 2 Cancer Research Laboratory Program, London Health Sciences Research Institute and Verspeeten Family Cancer Centre, London, ON Canada; 3 Department of Pathology and Laboratory Medicine, Schulich School of Medicine and Dentistry, Western University, London, ON Canada; 4 Children’s Health Research Institute, London Health Sciences Centre Research Institute, London, ON Canada; 5 Department of Paediatrics, Schulich School of Medicine and Dentistry, Western University, London, ON Canada; 6 Department of Oncology, Schulich School of Medicine and Dentistry, Western University, London, ON Canada

**Keywords:** Mechanisms of disease, Pancreatic cancer, Pancreatic cancer, Transcriptomics

## Abstract

Pancreatic ductal adenocarcinoma (PDAC) is the 3rd leading cause of cancer deaths in North America with ~12% survival 5 years after diagnosis. Risk factors for PDAC, including smoking and chronic pancreatitis, trigger the unfolded protein response (UPR). Global deletion of Activating Transcription Factor 3 (ATF3), a UPR mediator, restricts preneoplastic progression in mice expressing oncogenic KRAS (KRAS^G12D^). However, ATF3 is expressed in malignant and non-malignant cells suggesting it may affect multiple cell compartments in PDAC. Therefore, the goal of this study was to determine if ATF3 has epithelial-specific roles during PDAC initiation. Epithelial cells from mice expressing KRAS^G12D^ with (*Ptf1a*^*creERT/+*^*KRAS*^*G12D*/+^) or without ATF3 (*Atf3*^*−/−*^*Ptf1a*^*creERT/+*^*KRAS*^*G12D/+*^*; APK*) were characterized before and after pancreatic injury. Additionally, mice allowing acinar-specific *Atf3* deletion and KRAS^G12D^ expression (*A*^*acinar*^*PK*) were compared to *Ptf1a*^*creERT/+*^*KRAS*^*G12D*/+^ and *APK* mice following injury. RNA-seq revealed reduced oncogenic pathways in *APK* acinar cells consistent with reduced ADM formation in *APK* cultures. *Ptf1a*^*creERT/+*^*KRAS*^*G12D*/+^ and *APK* organoids showed differential gene expression and morphology, with *APK* organoids exhibiting reduced viability. In vivo, *APK* and *A*^*acinar*^*PK* tissue showed restricted neoplastic progression and KRAS signaling compared to *Ptf1a*^*creERT/+*^*KRAS*^*G12D*/+^ mice. This study indicates ATF3 works in a cell autonomous fashion, and its absence restricts *KRAS*^*G12D*^-mediated PDAC.

## Introduction

Pancreatic ductal adenocarcinoma (PDAC) is predicted to become the 2nd leading cause of cancer-related deaths by the year 2040 [1] with a 5-year survival rate of ~12% [2]. The low survival rate is due, in part, to late diagnosis and ineffective treatment options [3]. The most common driver mutations for PDAC are activating mutations of *KRAS*, with *KRAS*^*G12D*^ or *KRAS*^*G12V*^ mutations found in ~80% of all patients. However, *KRAS* mutations alone are not sufficient for causing pancreatic cancer, and additional events, such as mutations in *CDNK2A*, *SMAD4,* and *TP53* [4–7] or injury [8], are required for PDAC progression. Additionally, mouse models limiting activation of KRAS^G12D^ to adult pancreatic tissue require acute or repetitive pancreatic injury to promote progression beyond low-grade pancreatic intraepithelial neoplasia (PanIN; [9, 10]). In humans, hereditary or chronic forms of pancreatitis increase the risk factors for PDAC by 50 and 20-fold, respectively [11–14], suggesting injury triggers a molecular response that enhances KRAS^G12D^’s ability to promote progression to PDAC.

In response to injury, pancreatic acinar cells undergo acinar to duct cell metaplasia (ADM; [15]. Unresolved ADM, observed in chronic pancreatic injury, increases susceptibility for transformation to PanINs [16]. ADM is correlated with a rapid and sustained activation of stress pathways [17], inflammation [18], and hypoxia [19]. Central to this response is the unfolded protein response (UPR), which is activated by accumulation of unfolded or misfolded proteins in the endoplasmic reticulum (ER; [20]). The UPR suppresses general protein translation [21], increases protein folding capacity [22], and promotes protein degradation [23]. Due to high protein production in the pancreas, the UPR is actively maintained in acinar cells under physiological conditions [24]. However, several downstream mediators of the PERK pathway, including activating transcription factor 3 (ATF3), are expressed only after injury, suggesting a switch from physiological to pathological states in the UPR [17, 25].

ATF3 is a member of the activator protein-1 (AP-1) complex, which includes JUN, FOS, and other ATF family members [26, 27]. ATF3 regulates genes involved in cell cycle [28] and cell death [29] and can promote or repress transcription [30]. In cancer, ATF3’s role is context-dependent. ATF3 exerts anti-tumour effects in gastric cancer [26, 27] and tongue squamous cell carcinoma [31] by restricting Nrf2/Keap1 signaling or interferon-stimulating genes, respectively. However, in melanoma and non-small cell lung cancer, ATF3 has a pro-tumour role by promoting expression of PD-1L, which enables tumour cells to evade cytotoxic T-cells [32]. Previously, we showed global deletion of *Atf3* in mice restricted KRAS^G12D^’s ability to promote progression to high-grade PanIN lesions [33] and ATF3 targets genes that stabilize the mature acinar phenotype (*MIST1/BHLHA15*) or promote duct cell differentiation (*SOX9*) [34]. However, ATF3 is expressed in several cell types within PDAC tumours, including epithelial, immune, and fibroblast cell populations [33]. Therefore, it is unclear whether loss of ATF3 restricts PDAC progression through epithelial cell functions or through non-cell autonomous functions.

Two models were used to define the epithelial-specific contributions of ATF3 in mediating KRAS^G12D^’s effects during early stages of PDAC. First, epithelial cells were isolated from mice expressing KRAS^G12D^ with or without ATF3. Second, we generated a novel murine line allowing acinar cell-specific deletion of *Atf3* with *Kras*^*G12D*^ activation. In both models, reduced oncogenic signaling and PanIN progression were observed in the absence of ATF3. Interestingly, epithelial-specific loss of ATF3 had a more profound effect in restricting KRAS^G12D^-mediated neoplastic progression, suggesting ATF3 has multiple cell-specific roles in early PDAC progression.

## Results

**Fig. 1:**
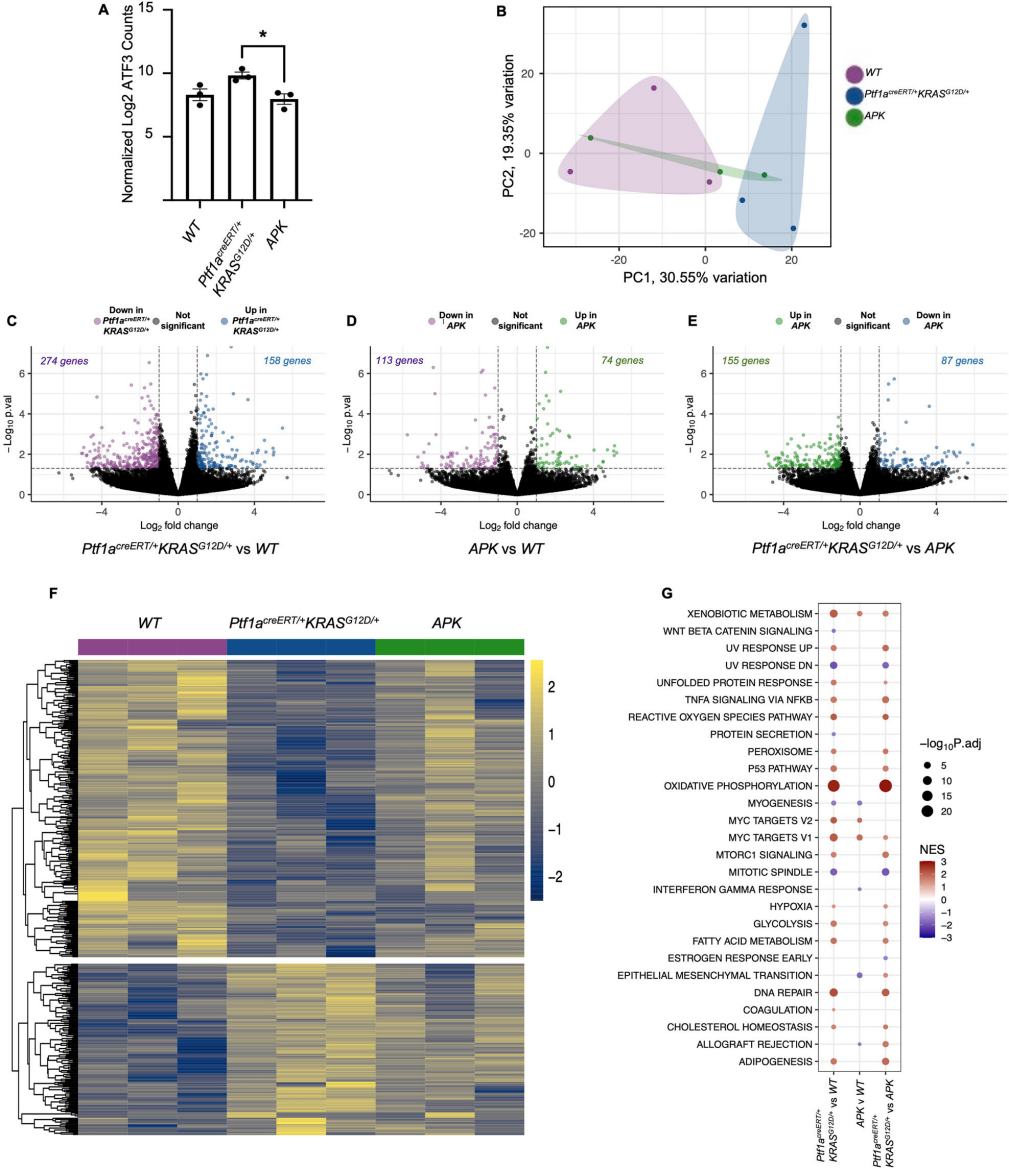
KRAS^G12D^–mediated transcriptional changes is reduced by the absence of ATF3. **A** Normalized expression of ATF3 in acinar cells isolated 22 days after tamoxifen-induction of KRAS^G12D^ with (*Ptf1a*^*creERT/+*^*KRAS*^*G12D/+*^) and without *(APK)* ATF3. Data is displayed as mean ± SEM (*n* = 3). One-way ANOVA with Tukey’s post hoc, *p < 0.05. **B** Principal component analysis clustering individual mice based on RNA-seq analysis. Each point represents an individual mouse. **C–E** Volcano plots showing differentially expressed genes (DEGs) between **C** *Ptf1a*^*creERT/+*^*KRAS*^*G12D/+*^ and wild type acini, **D** *APK* and wild type acini, or **E** *Ptf1a*^*creERT/+*^*KRAS*^*G12D/+*^ and *APK* acini (*n* = 3 for each genotype). **F** Heat map showing DEGs between *Ptf1a*^*creERT/+*^*KRAS*^*G12D/+*^ and wild type acini. Expression is shown for *APK* acini as well. **G** Gene set enrichment analysis identifying pathways that are differentially enriched between at least two genotypes based on RNA-seq analysis. NES normalized enrichment score.

Since ATF3 is a transcription factor, the effects of deleting ATF3 in *KRAS*^*G12D*^-expressing epithelial cells was first examined at the transcriptomic level. RNA-seq analysis was performed on whole pancreatic tissue or isolated acinar cells from control C57Bl/6 (wild type), *Ptf1a*^*creERT/+*^*KRAS*^*G12D/+*^, and *APK* mice 22 days after induction of KRAS^G12D^. *Ptf1a*^*creERT/+*^*KRAS*^*G12D/+*^ and *APK* mice showed no significant changes in weight (Supplemental Fig. S1A) or pancreatic morphology (Supplemental Fig. S1B) when compared to wild type tissue. As previously observed, *Atf3* expressed was negligible in bulk tissue, but significantly increased in all genotypes upon acinar cell isolation, consistent with *Atf3* expressed only after injury [17, 34]. RNA sequencing confirmed targeted deletion of exon 2 in *APK* acini (Supplemental Fig. S1C), which includes the translational start site for ATF3. Interestingly, *Ptf1a*^*creERT/+*^*Kras*^*G12D/+*^ acini showed significantly higher levels of *ATF3* expression compared to *APK* or wild type acini (Fig. 1A), suggesting KRAS^G12D^ and injury have an additive effect on ATF3 expression. Since expression of ATF3 is limited in bulk pancreatic tissue, analysis focused on isolated acinar cells.

Principal component analysis (PCA; *n* = 3/genotype) showed distinct clustering of wild type and *Ptf1a*^*creERT/+*^*Kras*^*G12D/+*^ groups based on RNA-seq of isolated acinar cells. *APK* samples did not cluster distinctly from either group (Fig. 1B). Pair-wise comparison of transcriptomes identified 432 differentially expressed genes (DEGs) between *Ptf1a*^*creERT/+*^*Kras*^*G12D/+*^ and wild type acini (log2 fold change <−1 and >1; *p*-value < 0.05; Fig. 1C and Supplemental Table S1). The absence of ATF3 significantly reduced this response with only 187 DEGs between *APK* and wild type acini, 56.7% fewer differences compared to *Ptf1a*^*creERT/+*^*Kras*^*G12D/+*^ mice (Fig. 1D and Supplemental Table S2). Direct comparison of *Ptf1a*^*creERT/+*^*Kras*^*G12D/+*^ and *APK* transcriptomes revealed 242 DEGS (Fig. 1E).

To determine if *Atf3* deletion affected KRAS^G12D^’s impact on the transcriptome, we examined whether DEGs (*p*-value < 0.05) between wild type and *Ptf1a*^*creERT/+*^*Kras*^*G12D/+*^ acini were also altered between wild type and *APK* acini. In total, 78.2% of all DEGs between *Ptf1a*^*creERT/+*^*Kras*^*G12D/+*^ and WT acini did not reach statistical significance in differential expression between wild type and *APK* acini (Fig. 1F and Supplemental Table S1). These ATF3-dependent changes included 108 DEGs with higher and 230 DEGs with lower expression in *Ptf1a*^*creERT/+*^*Kras*^*G12D/+*^ acini compared to the wild type acinar transcriptome (Fig. 1F). Several AP-1 family members enhanced by KRAS^G12D^, including *Fos* and *JunB*, and stress response genes, including *Egr2*, *Ier2*, and *Ier3*, were not altered in the absence of ATF3.

Gene set enrichment analysis (GSEA) comparing *Ptf1a*^*creERT/+*^*Kras*^*G12D/+*^ to wild type acini, identified increased enrichment for several processes previously linked to ATF3, including hypoxia, the UPR, and ROS signaling, and oncogenic processes including TNFα and P53 signaling (Fig. 1G). Similar comparisons between *APK* and wild type acini revealed none of these gene sets were enriched, supporting a role for ATF3 in KRAS^G12D^’s ability to initiate cancer progression (Fig. 1G). Direct comparison between *Ptf1a*^*creERT/+*^*Kras*^*G12D/+*^ and APK acini confirm reduced enrichment for UPR, ROS, and hypoxia pathways in APK acini (Supplemental Fig. S2). GSEA comparing *APK* to wild type acini identified few pathways enriched specifically in APK acini (Fig. 1G), but pathways for epithelial mesenchymal transition, DNA repair and metabolism were either negatively enriched or not enriched in APK compared to either *Ptf1a*^*creERT/+*^*Kras*^*G12D/+*^ to wild type acini.

**Fig. 2:**
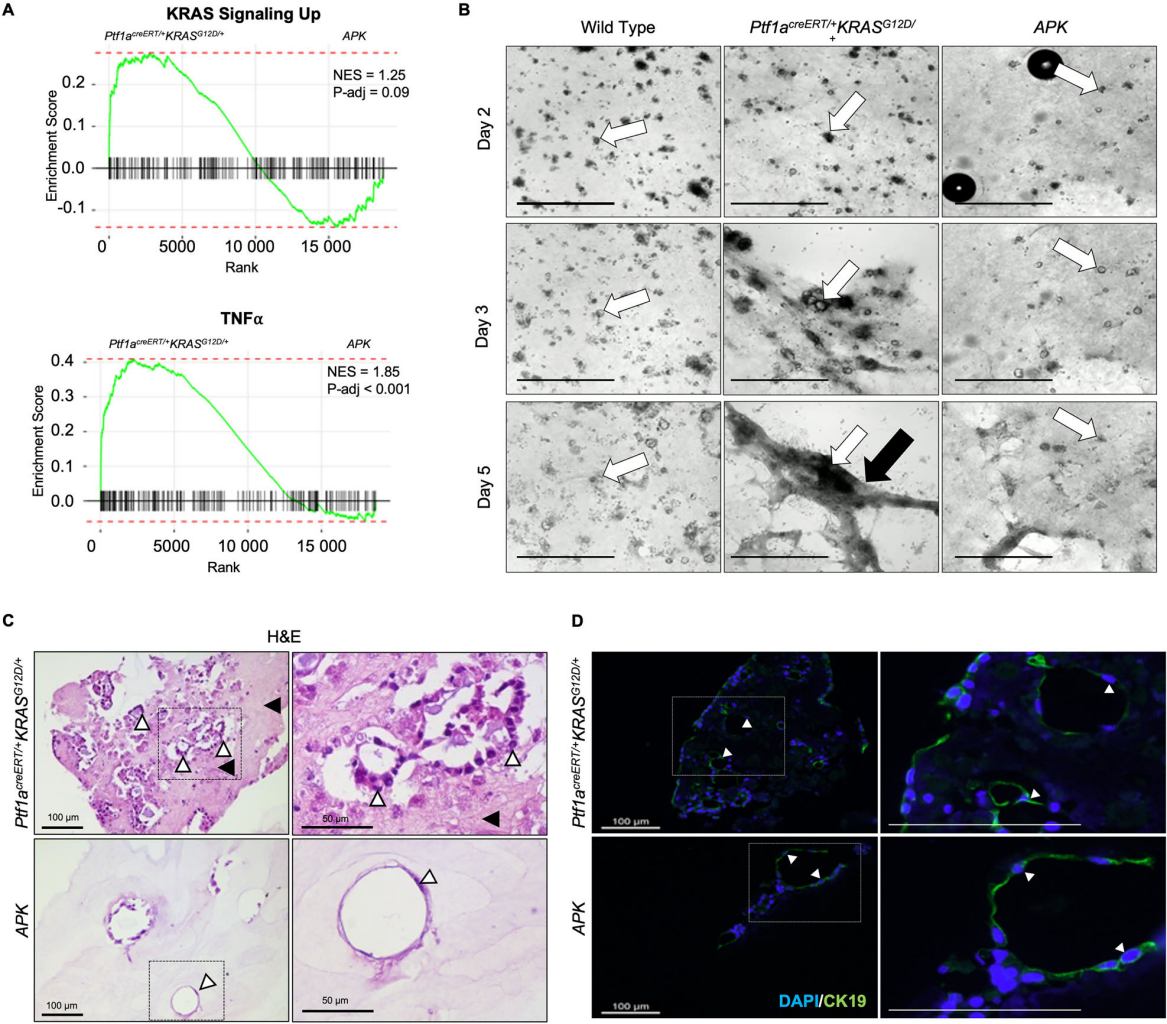
KRAS^G12D^-mediated pathways and phenotypes are restricted by germline deletion of *Atf3.* **A** Gene set enrichment analysis comparing enrichment of “KRAS signaling up” and “TNFα” KEGG pathways between *Ptf1a*^*creERT/+*^*KRAS*^*G12D/+*^ and *APK* acini isolated 22 days after KRAS^G12D^ activation. NES, normalized enrichment score. **B** Representative phase contrast images of acini from wild type, *Ptf1a*^*creERT/+*^*KRAS*^*G12D/+*^, and *APK* mice grown in collagen for 1, 3, or 5 days. White arrows identify the same acinar cluster over time. Black arrow indicates matrix accumulation. See Movies 1–3 for the complete time course. Scale bar = 100 µm. **C** Representative H&E-stained sections from 5 day *Ptf1a*^*creERT/+*^*KRAS*^*G12D/+*^ and *APK* cultures. White arrowheads indicate duct like cells. Black arrowheads indicate matrix. **D** Immunofluorescent staining for CK19 (green) in *Ptf1a*^*creERT/+*^*KRAS*^*G12D/+*^ and *APK* cultures. White arrowheads indicate CK19+ cells. Scale bar = 100 µm. Each experiment was carried out three times with different mice from each genotype.

Reduced KRAS and TNFα signalling in the absence of ATF3 (Fig. 2A and Supplemental Fig. S3) should correlate to reductions in acinar to duct cell metaplasia (ADM; [35, 36]. To assess ADM, acinar cells from wild type, *Ptf1a*^*creERT/+*^*KRAS*^*G12D/+*^, or *APK* pancreata were cultured in collagen and assessed over five days. Upon isolation, acini from all genotypes were phenotypically similar (Fig. 2B). By day 3, ADM was observed in the majority of *Ptf1a*^*creERT/+*^*KRAS*^*G12D/+*^ acini, while wild type and *APK* cultures showed very few ADM structures. By day 5, cyst-like structures were apparent in some wild type (Movie 1) or *APK* (Movie 2) acini, while most of the *Ptf1a*^*creERT/+*^*KRAS*^*G12D/+*^ acini appeared to embed into collagen accumulations that form during culture (Fig. 2B and Movie 3). H&E staining of sectioned cultures at day 5 confirmed many ADM-like clusters in *Ptf1a*^*creERT/+*^*KRAS*^*G12D/+*^ cultures with only small, simple epithelial structures rarely observed in *APK* cultures (Fig. 2C). Staining also revealed eosinophilic matrices surrounding *Ptf1a*^*creERT/+*^*KRAS*^*G12D/+*^ ADM. IF staining for CK19 confirmed duct-like cells in *Ptf1a*^*creERT/+*^*KRAS*^*G12D/+*^ and *APK* cultures, indicative of ADM (Fig. 2D). These results support an epithelial-specific role for ATF3 in enhancing KRAS^G12D^’s ability to initiate ADM.

**Fig. 3:**
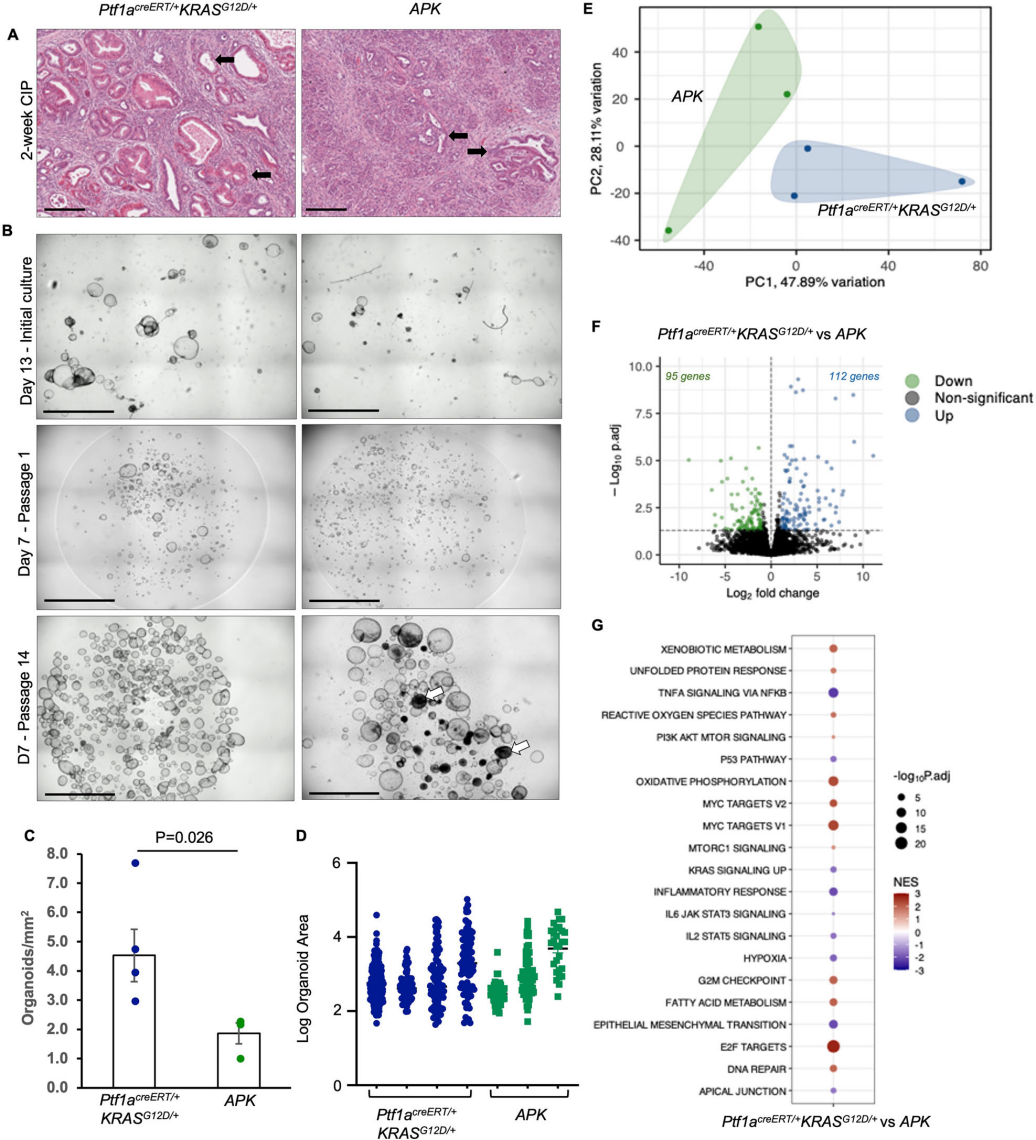
Loss of ATF3 reduces but does not prevent organoid generation from neoplastic tissue, but alters the KRAS transcriptional program. **A** Representative H&E staining from *Ptf1a*^*creERT/+*^*KRAS*^*G12D/+*^ and *APK* mice 2 weeks after cerulein treatment. Arrows indicate duct structures (*n* = 6/genotype). Scale bars = 200 µm. **B** Representative brightfield images of organoids 13 days after initial isolation, seven days after first passage, or seven days after consecutive passaging. White arrows indicate morphologically distinct organoids. Scale bars = 2.4 mm. **C** Quantification of organoids formed after isolation from *n* = 4 *Ptf1a*^*creERT/+*^*KRAS*^*G12D/+*^ and *n* = 3 *APK* mice 2 weeks after cerulein treatment. Data presented as mean ± SEM. Statistical analysis by *t*-test. **D** Quantification of organoid size 13 days after isolation from *Ptf1a*^*creERT/+*^*KRAS*^*G12D/+*^ (*n* = 4) and *APK* (*n* = 3) mice 2 weeks after cerulein treatment. Data presented as mean ± SEM. **E** Principal component analysis plot shows no overlap between *Ptf1a*^*creERT/+*^*KRAS*^*G12D/+*^ and *APK* organoids (*n* = 3/genotype). **F** Volcano plot showing DEGs between *Ptf1a*^*creERT/+*^*KRAS*^*G12D/+*^ and *APK* mice. **G** Dot plot for gene set enrichment analysis comparing RNA-sequencing between *Ptf1a*^*creERT/+*^*KRAS*^*G12D/+*^ and *APK* organoids. NES normalized enrichment score.

Our previous studies suggested ATF3 also has a role in maintaining PanIN lesions. To examine an epithelial-specific role for ATF3 post-ADM, we established organoid cultures 14 days after cerulein treatment from *Ptf1a*^*creERT/+*^*KRAS*^*G12D/+*^ and *APK* tissue. At this time point, *APK* and *Ptf1a*^*creERT/+*^*KRAS*^*G12D/+*^ tissues show extensive ADM (Fig. 3A). Upon isolation, *APK* cultures resulted in only 41.2% of the organoids that appeared in *Ptf1a*^*creERT/+*^*KRAS*^*G12D/+*^ cultures (*p* < 0.05; *n* = 4 and 3 for *Ptf1a*^*creERT/+*^*KRAS*^*G12D/+*^ and *APK* organoid lines, respectively; Fig. 3B top panel, C). However, the organoids that did form in *APK* cultures were comparable in size to *Ptf1a*^*creERT/+*^*KRAS*^*G12D/+*^ organoids (Fig. 3D). Upon passaging, *APK* cultures showed similar numbers of organoids to *Ptf1a*^*creERT/+*^*KRAS*^*G12D/+*^ cultures, suggesting selection of cells that could be expanded in the absence of ATF3 (Fig. 3B, middle panel). In total, organoid lines (Fig. 3B, bottom panel) were developed from all *APK* and *Ptf1a*^*creERT/+*^*KRAS*^*G12D/+*^ isolation performed, with 6 distinct organoid lines developed for each genotype and included both male and female lines. To validate that organoid lines are derived from transformed PanIN cells and not normal ductal epithelial cells, RT-PCR followed by Sanger sequencing of KRAS mRNA was performed to confirm the presence of mutant *KRAS*^*G12D*^ and acinar cell origin for all organoid lines established (Supplemental Fig. S4). In all lines, at least 50% of the cDNA product contained a T to C change at codon 12, confirming biallelic expression of wild type and mutated *KRAS* RNA.

To determine whether the absence of ATF3 altered gene expression in these organoid lines, we performed RNA-seq on three *APK* and three *Ptf1a*^*creERT/+*^*KRAS*^*G12D/+*^ lines. PCA showed marginal separation of organoids based on genotype (Fig. 3E). However, DESeq2 identified 207 DEGs between *Ptf1a*^*creERT/+*^*KRAS*^*G12D/+*^ and *APK* organoids (*n* = 3; log2FC <−1 and >1; padj < 0.05; Fig. 3F and Supplemental Table S3). GSEA identified enrichment for stress-related pathways such as DNA repair, ROS signaling, and the UPR in *Ptf1a*^*creERT/+*^*KRAS*^*G12D/+*^ organoids (Fig. 3G), similar to pathways altered in acinar cells. Surprisingly, *APK* organoid transcriptomes were enriched for genes involved in TNFα, P53, and KRAS signaling (Fig. 3G), differing from pathway analysis in acinar cells.

**Fig. 4:**
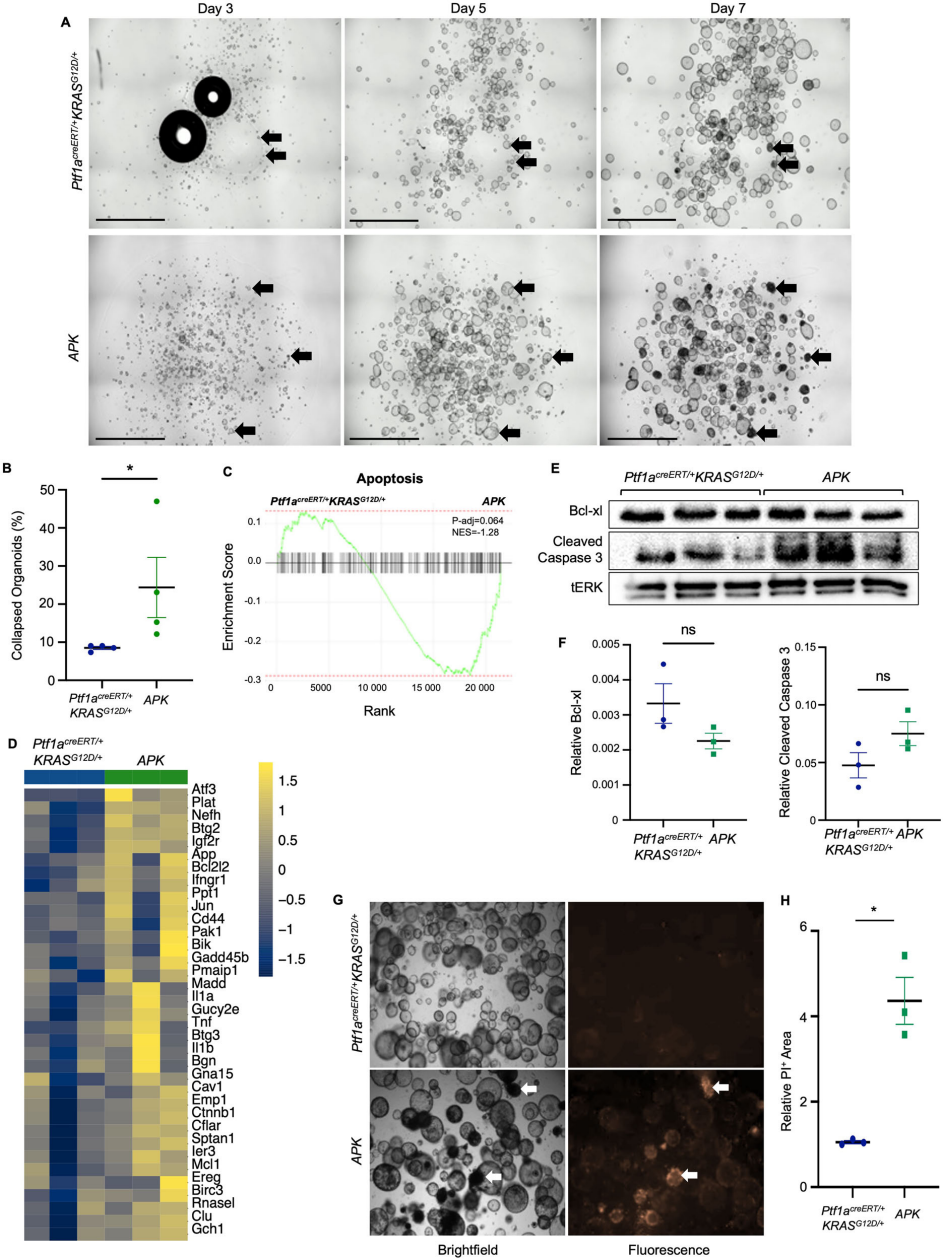
Loss of ATF3 increases cell death in organoids cultured in 3D inclusion model. **A** Representative brightfield images of organoids cultured in inclusion model 3, 5, and 7 days after passaging. *Ptf1a*^*creERT/+*^*KRAS*^*G12D/+*^ and *APK* organoid cultures are similar at day 3 with small organoids beginning to form. By day 7, APK cultures show more dark and collapsed organoids compared to the cyst-like organoids seen in *Ptf1a*^*creERT/+*^*KRAS*^*G12D/+*^ cultures. Black arrows indicate individual organoids over time. Magnification bar = 2.4 mm. **B** Quantification of collapsed organoids in *Ptf1a*^*creERT/+*^*KRAS*^*G12D/+*^ and *APK* cultures 7 days after passage displayed as mean ± SEM. *N* = 4; *P < 0.05. **C** GSEA plot comparing RNA from *Ptf1a*^*creERT/+*^*KRAS*^*G12D/+*^ and *APK* cultures shows enrichment of apoptosis gene set in APK organoids compared to *Ptf1a*^*creERT/+*^*KRAS*^*G12D/+*^ organoids. NES, normalized enrichment score. **D** Heat map for differentially expressed genes in apoptosis gene signature. **E** Western blots on protein extracted from 3 independent organoid lines 7 days after plating and examined for Bcl-xL, cleaved caspase 3, and tERK. **F** Densitometric analysis of Bcl-xL and cleaved caspase 3 western blots. Each dot represents a different organoid line. ns, not significant. **G** Representative brightfield and fluorescent images of *Ptf1a*^*creERT/+*^*KRAS*^*G12D/+*^ and *APK* organoid cultures stained with propidium iodide. Propidium iodide (red) positively stains dark and collapsed organoids (arrows). Magnification bar = 1 mm. **H** Quantification of PI staining in *Ptf1a*^*creERT/+*^*KRAS*^*G12D/+*^ and *APK* cultures 7 days after passage displayed as mean ± SEM. Statistical analysis by *t*-test with *n* = 4, *, *P* < 0.05.

To determine if these differences in pathway enrichment resulted in functional changes, we examined *Ptf1a*^*creERT/+*^*KRAS*^*G12D/+*^ and *APK* organoids in two different culture conditions. The inclusion model [37], which seeds cells in matrigel domes (Fig. 4 and Movies 4 and 5), resulted in *Ptf1a*^*creERT/+*^*KRAS*^*G12D/+*^ and *APK* organoids developing into cyst-like structures within 5 days of passage (Fig. 4A). Starting at day 5, *APK* organoids appear to collapse into smaller, denser structures (Fig. 4A and Movie 5). While collapsing organoids were apparent in *Ptf1a*^*+/creERT*^*Kras*^*G12D/+*^ cultures, these collapsing organoids appeared later, typically after day 7, and were fewer in number. Quantification at day seven showed a higher percentage (*p* = 0.02) of collapsed organoids in *APK* cultures (24% ± 7.8%) compared to *Ptf1a*^*+/creERT*^*Kras*^*G12D/+*^ cultures (9% ± 0.4%, Fig. 4B; *n* = 4 for each genotype). While GSEA identified increased enrichment for apoptotic-related genes in *APK* cultures (Fig. 4C, D), suggesting collapsed organoids represent apoptotic cells, western blot analysis for pro-, cleaved caspase 3, and anti-apoptotic markers, Bcl-xL, were inconsistent between organoid lines (Fig. 4E, F). *APK* cultures showed modest decreases in Bcl-xL and increases in cleaved caspase 3 compared to *Ptf1a*^*+/creERT*^*Kras*^*G12D/+*^ cultures, but these differences did not reach significance (Fig. 4F). An alternative examination of cell death involving the uptake of propidium iodide showed few *Ptf1a*^*+/creERT*^*Kras*^*G12D/+*^ organoids stained for propidium iodide (Fig. 4G, H). Conversely, a fourfold increase in propidium iodide positive cells was observed in *APK* organoids, and confirmed collapsed organoids consisted mostly of dying cells (Fig. 4G, H). This increased cell death was also reflected by a reduction in the number of organoids in *APK* cultures at day seven (Supplemental Fig. S4B).

**Fig. 5:**
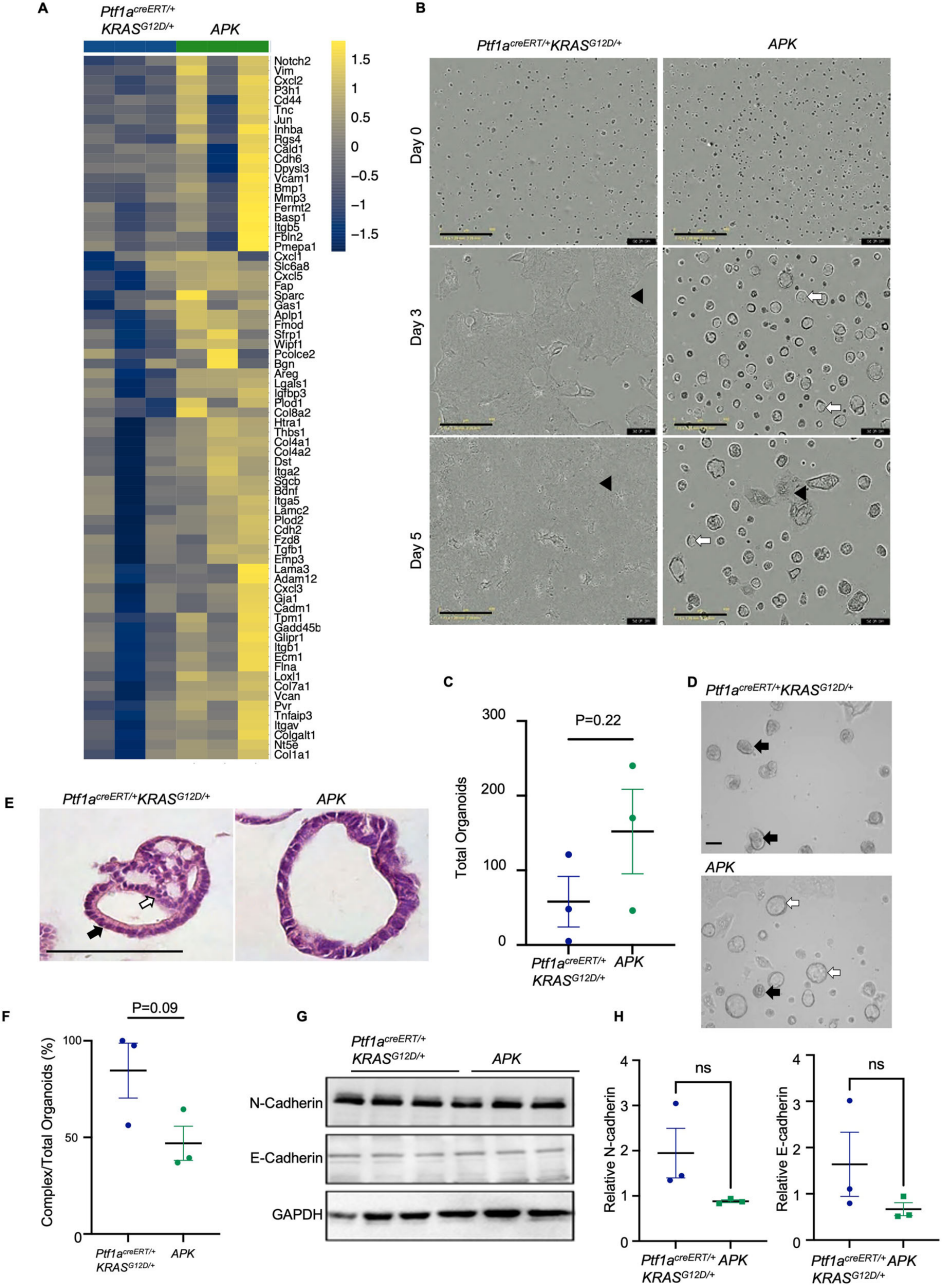
Loss of ATF3 affects growth of cells grown in an organotypic culture. **A** Heat map for differentially expressed genes between *Ptf1a*^*creERT/+*^*KRAS*^*G12D/+*^ and *APK* organoids based on RNA-seq developed from inclusion cultures. Each lane represents a different organoid line. **B** Capture images from movies depicting 2D cell attachment in *Ptf1a*^*creERT/+*^*KRAS*^*G12D/+*^ and *APK* cultures. Scale bar = 400 µm. Black arrowheads show areas of 2D attachment. White arrows show cyst-like organoids. **C** Quantification of total organoids observed in *Ptf1a*^*creERT/+*^*KRAS*^*G12D/+*^ and *APK* cultures 5 days after passage displayed as mean ± SEM. *P*-value is shown, *n* = 3. **D** Representative brightfield organoids remaining 5 days after passaging. Black arrows indicate organoids with a complex structure, while white arrows indicate cyst-like organoids. Magnification bar = 200 µm. **E** Representative H&E staining of organoids at Day 5 in culture showing complex organoids from *Ptf1a*^*creERT/+*^*KRAS*^*G12D/+*^ cultures and cyst-like organoids from *APK* cultures. Black arrow indicate columnar epithelium, while white arrow indicate stratified epithelium. Magnification bars = 100 µm. **F** Quantification of the number of complex organoids observed in *Ptf1a*^*creERT/+*^*KRAS*^*G12D/+*^ and *APK* cultures 5 days after passage displayed as mean ± SEM. Statistical analysis by *t*-test. *N* = 3, *P*-value is indicated. **G** Western blot analysis of N-cadherin, E-cadherin, or GAPDH (control) on protein isolated from *Ptf1a*^*creERT/+*^*KRAS*^*G12D/+*^ and *APK* organotypic cultures 5 days after plating. Each lane represents a different organoid line. **H** Quantification of N-cadherin or E-cadherin relative to GAPDH levels. Data displayed as mean ± SEM. Statistical analysis by Welch’s *t*-test. ns not significant.

Since pathway analysis suggested the epithelial to mesenchymal transition (EMT) pathway may be enriched in *APK* organoids (Figs. 3G and 5A), the growth of *Ptf1a*^*+/creERT*^*Kras*^*LSL-G12D/+*^ and *APK* organoids was compared in an organotypic culture model, with cells seeded on a layer of Matrigel (Zeeberg et al., [38]). Surprisingly, despite being enriched for markers of EMT (Fig. 5A), *APK* cultures were predominantly maintained as 3D organoids with limited areas of 2D morphology (Fig. 5B and Movie 7). Conversely, *Ptf1a*^*creERT/+*^*KRAS*^*G12D/+*^ cultures showed a rapid loss of a 3D morphology and often grew as monolayers (Fig. 5B and Movie 6). By Day 5, *Ptf1a*^*creERT/+*^*KRAS*^*G12D/+*^ cultures showed a confluent monolayer with few organoids maintained as 3D structures. Continued growth of *Ptf1a*^*creERT/+*^*KRAS*^*G12D/+*^ cells was readily apparent in 2D (Movie 6), while *APK* cells that took on a 2D morphology did not continue to grow (Movie 7). By day 5, *Ptf1a*^*creERT/+*^*KRAS*^*G12D/+*^ cultures trended towards having fewer organoids than *APK* cultures, likely due to their conversion to a 2D morphology (Fig. 5C). Even those organoids retained in culture showed different morphologies between genotypes. *Ptf1a*^*creERT/+*^*Kras*^*G12D/+*^ organoids often appeared with a more complex architecture while *APK* organoids typically displayed a cyst-like morphology similar to that observed in inclusion cultures (Fig. 5D). H&E staining of *Ptf1a*^*creERT/+*^*KRAS*^*G12D/+*^ organoids revealed stratified (white arrow) and columnar epithelium (black arrow) consistent with progressed PanIN subtypes (Fig. 5E), while *APK* organoids were mostly composed of simple epithelium (Fig. 5E). Quantification comparing the number of organoids based on morphology suggested more *Ptf1a*^*creERT/+*^*KRAS*^*G12D/+*^ organoids took on this complex morphology, although the numbers did not reach significant (Fig. 5F; *p* < 0.09, *n* = 3).

To assess whether there was a difference in definitive EMT markers, western blot analysis was performed for N-cadherin and E-cadherin (Fig. 5G, H). Consistent with morphological analysis, N-cadherin levels were higher, although not significant (*p* > 0.05; *n* = 3), in *Ptf1a*^*creERT/+*^*Kras*^*G12D/+*^ organoid cultures, while E-cadherin levels showed no difference between genotypes. These results suggest transcriptomic analysis reflects potential differences in gene expression compensation with the absence of ATF3, dysregulating gene expression of pathways involved in pancreatic cancer progression.

**Fig. 6:**
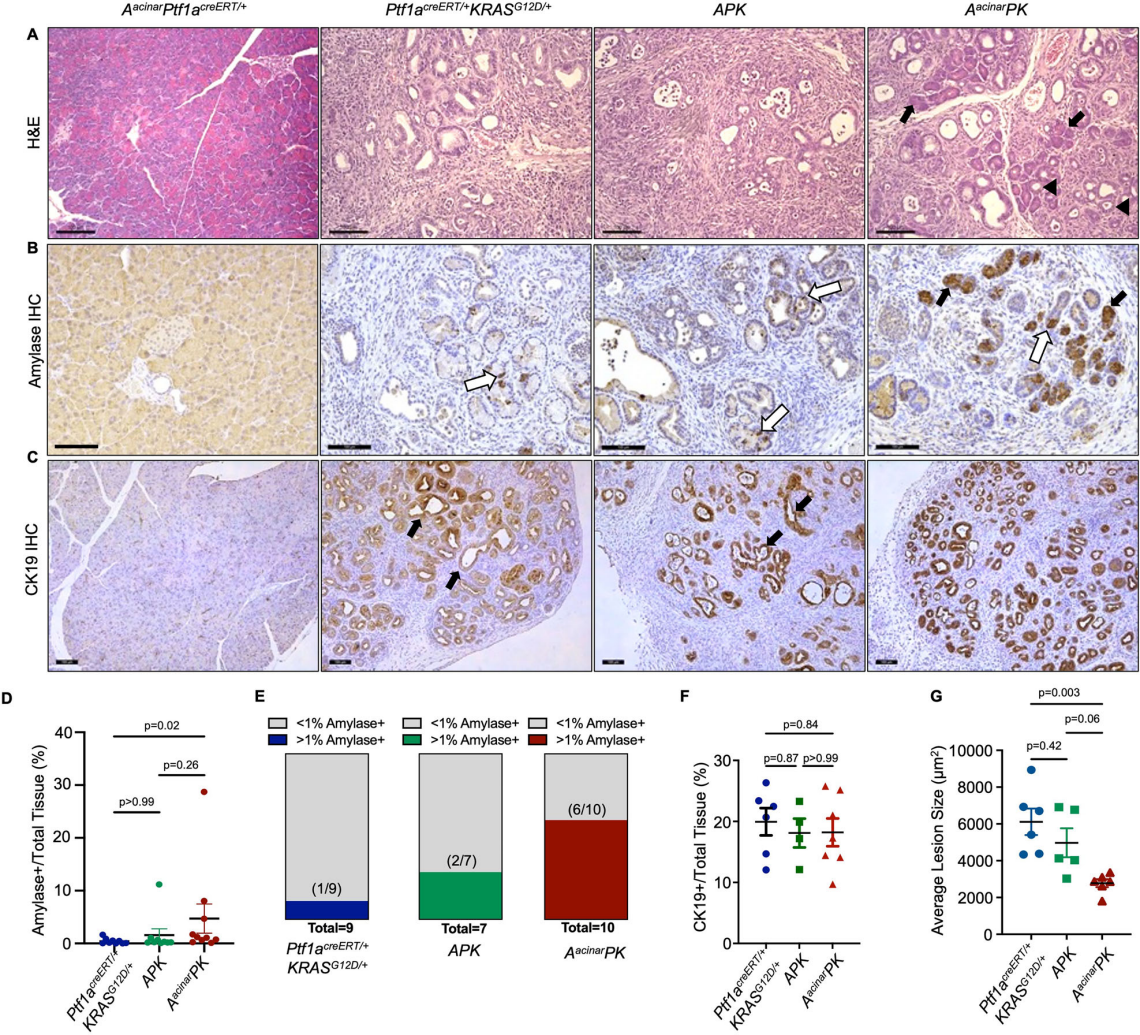
Acinar specific loss of ATF3 reduces PanIN lesion progression in vivo. **A** Representative H&E staining of pancreatic tissue sections from *A*^*acinar*^*Ptf1a*^*creERT/+*^*, Ptf1a*^*creERT/+*^*KRAS*^*G12D/+*^, *APK*, and *A*^*acinar*^*PK* mice 2 weeks after CIP treatment. *A*^*acinar*^*PK* tissue display pockets of acinar tissue (black arrows) and some ADM (arrowheads). Magnification bars = 100 µm. **B** Representative IHC staining for amylase shows individual positive cells within putative ADM of *Ptf1a*^*creERT/+*^*KRAS*^*G12D/+*^ and *APK* tissue (white arrows). *A*^*acinar*^*PK* mice maintain pockets of amylase+ acini (black arrows). **C** Representative IHC staining for CK19 reveals large CK19+ ducts in *Ptf1a*^*creERT/+*^*KRAS*^*G12D/+*^ and *APK* tissue, which is not observed in *A*^*acinar*^*PK* tissue. Quantification of **D** amylase+ area displayed as mean ± SEM or **E** the number of mice from each genotype that maintain amylase+ tissue. *N* = 9, 9, and 10 for *Ptf1a*^*creERT/+*^*KRAS*^*G12D/+*^*, APK*, and *A*^*acinar*^*PK* mice, respectively. Statistical analysis by Kruskal–Wallis test. **F** Quantification of CK19+ area displayed as mean ± SEM. *N* = 6, 4, and 7 for *Ptf1a*^*creERT/+*^*KRAS*^*G12D/+*^*, APK*, and *A*^*acinar*^*PK* mice, respectively. Statistical analysis by one-way ANOVA with Tukey’s post hoc. **G** Quantification of CK19+ lesion size displayed as mean ± SEM. *N* = 6, 5, and 6 for *Ptf1a*^*creERT/+*^*KRAS*^*G12D/+*^*, APK*, and *A*^*acinar*^*PK* mice, respectively. Statistical analysis by one-way ANOVA with Tukey’s post hoc. In all cases, *P* values are shown.

It is possible external cues from the *APK* environment influence epithelial cell differentiation even after isolation. Also, the germ line deletion of *Atf3* may underlie some of the potential compensation observed in cultures. Therefore, we generated an inducible, acinar-specific knockout for *Atf3* in the KRAS^G12D^ background (*Atf3*^*fl/fl*^*Ptf1a*^*creERT/+*^*Kras*^*G12D/+*^; referred to as *A*^*acinar*^*PK*; Supplemental Fig. S5A). Deletion of exon 2 and the *Atf3* translational start site was confirmed 21 days after tamoxifen in *A*^*acinar*^*PK* and *Ptf1a*^*creERT/+*^*Atf3*^*fl/fl*^ acinar tissue (Supplemental Fig. S5B) and confirmed loss of ATF3 through western blot analysis on protein extracts isolated 4 h into cerulein treatment, a time point where ATF3 is expressed to high levels [34] (Supplemental Fig. S5B). We also confirmed the genomic region containing exon 2 of the *Atf3* gene was deleted in RNA isolated from acinar cell cultures 20 days after tamoxifen treatment (Supplemental Fig. S5C). Ten days after tamoxifen treatment, acute pancreatic injury was induced in *Ptf1a*^*creERT/+*^*KRAS*^*G12D/+*^, *APK*, and *A*^*acinar*^*PK* mice with cerulein (Supplemental Fig. S6A). No significant differences in body weight were observed between groups regardless of genotype or treatment (Supplemental Fig. S6B, C), with all groups showing reduced weight upon treatment. Consistent with previous studies, mice expressing KRAS^G12D^ begin to show weight loss by two weeks at which time mice were sacrificed. Pancreatic to body weight ratios were increased in *Ptf1a*^*creERT/+*^*KRAS*^*G12D/+*^ (*n* = 9), *A*^*acinar*^*PK* (*n* = 8), and *APK* (*n* = 8) mice, although only *Ptf1a*^*creERT/+*^*KRAS*^*G12D/+*^ were significantly different than WT or *A*^*acinar*^*P* mice (*p* < 0.05; Supplemental Fig. S6D). H&E staining of pancreatic tissue revealed extensive, high-grade PanIN lesions and fibrosis with no observable acinar tissue in cerulein-treated *Ptf1a*^*creERT/+*^*KRAS*^*G12D/+*^ mice (Fig. 6A), while *A*^*acinar*^*PK* pancreata retained some acinar tissue and lesions appeared to be limited to ADM and low-grade PanINs. Global loss of ATF3 resulted in high-grade lesions similar to that observed in *Ptf1a*^*creERT/+*^*KRAS*^*G12D/+*^ mice (Fig. 6A), mirroring earlier studies at this time point [33]. Histology on saline-treated mice showed no differences in overall morphology (Supplemental Fig. S6E).

Immunohistochemistry (IHC) for amylase (acinar cell marker; Fig. 6B, D, E) revealed clusters of acinar cells in *A*^*acinar*^*PK* mice, while amylase expression was limited to small groups of cells in putative ADM or PanIN lesions of *Ptf1a*^*creERT/+*^*KRAS*^*G12D/+*^ and *APK* tissues (Fig. 6B, D). Most *A*^*acinar*^*PK* mice (6/10; 4.7 ± 2.7% of the tissue) retained acinar tissue, while only 2/7 *APK* (1.6 ± 1.2% of tissue; and 1/9 *Ptf1a*^*creERT/+*^*KRAS*^*G12D/+*^ (0.4 ± 0.2% of tissue) mice showed any amylase+ tissue (Fig. 6E). IHC for the duct cell marker cytokeratin 19 (Fig. 6C) showed no difference in the amount of CK19+ area (Fig. 6F) between genotypes, and lesion size (Fig. 6G) was not different between *APK* and *Ptf1a*^*creERT/+*^*KRAS*^*G12D/+*^ mice (*p* = 0.41), consistent with previous data [33]. However, CK19+ lesions in *A*^*acinar*^*PK* mice were significantly smaller (*n* = 6) compared to *Ptf1a*^*creERT/+*^*KRAS*^*G12D/+*^ (Fig. 6G, *n* = 6; *p* < 0.01). The number of CK19+ lesions was higher in *A*^*acinar*^*PK* tissue, which may reflect reduced progression to larger PanINs in these mice (Supplemental Fig. S6F).

**Fig. 7:**
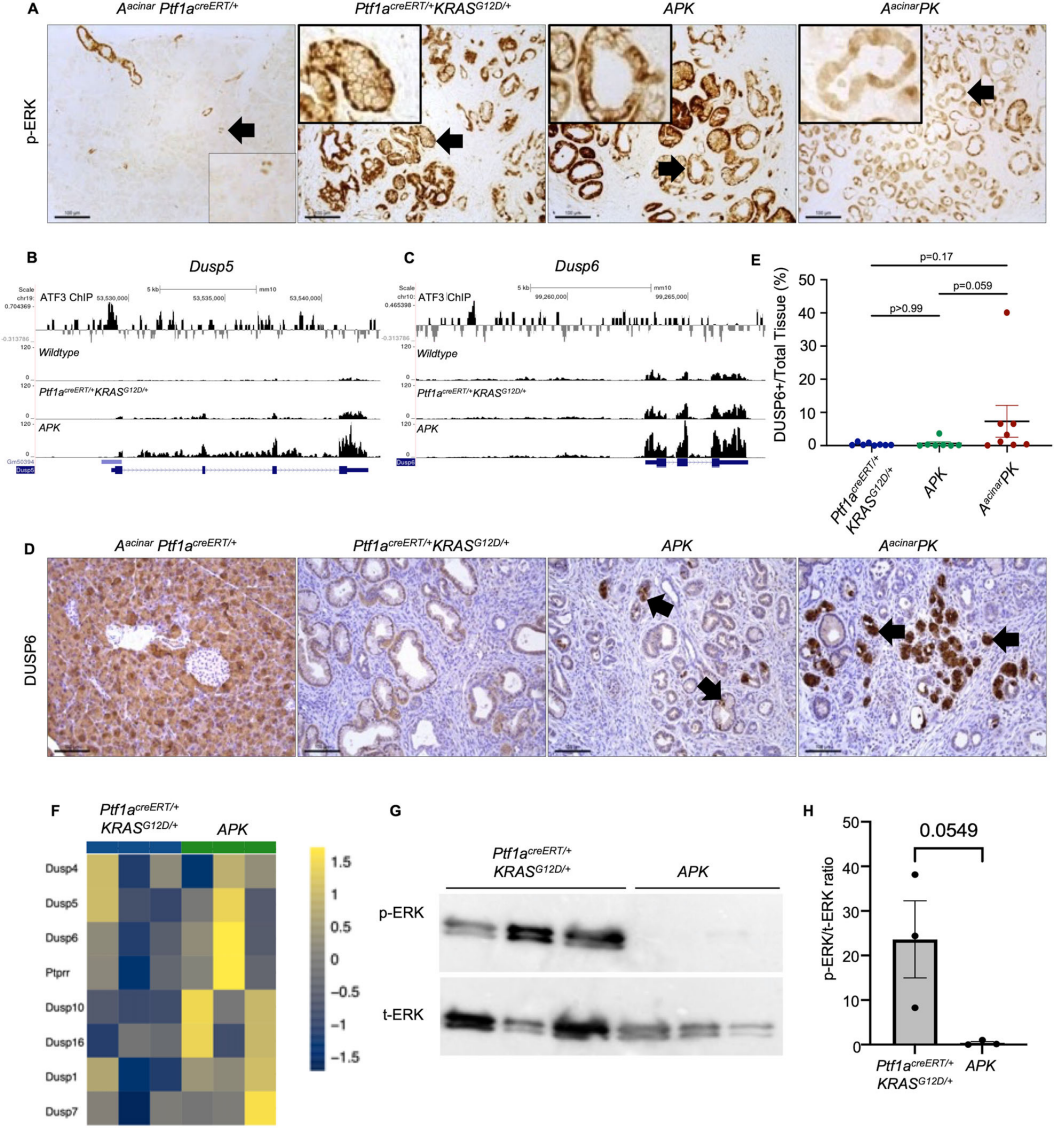
Epithelial-specific deletion of ATF3 decreases MAPK signaling following activation of KRAS^G12D^ and pancreatic injury. **A** Representative IHC staining for phospho (p) ERK 2-weeks in *A*^*acinar*^*Ptf1a*^*creERT/+*^*, Ptf1a*^*creERT/+*^*KRAS*^*G12D/+*^, *APK*, and *A*^*acinar*^*PK* mice after CIP treatment. *Ptf1a*^*creERT/+*^*KRAS*^*G12D/+*^ and *APK* mice display widespread nuclear and cytoplasmic staining in PanIN lesions while *A*^*acinar*^*PK* mice show diffuse cytoplasmic localization with nuclear localization limited to a few cells in PanIN lesions. Insets show magnified individual organoids indicated by arrows. **B**, **C** Gene tracks displaying **B** *Dusp5* and **C** *Dusp6* expression in wild type, *Ptf1a*^*creERT/+*^*KRAS*^*G12D/+*^, and *APK* tissue 22 days after KRAS^G12D^ induction, or ATF3 enrichment based on ChIP-seq from Fazio et al. [34]. **D** Representative IHC staining 2-weeks after CIP treatment for DUSP6, a negative regulator of KRAS signaling. Little to no positive staining was observed in PanIN lesions of *Ptf1a*^*creERT/+*^*KRAS*^*G12D/+*^ tissue, while *APK* and *A*^*acinar*^*PK* tissue showed some positive staining throughout the tissue. Magnification bars = 100 µm. **E** Quantification of DUSP6 staining displayed as mean ± SEM. *N* = 8, 7, and 8 for *Ptf1a*^*creERT/+*^*KRAS*^*G12D/+*^*, APK*, and *A*^*acinar*^*PK* mice, respectively. Statistical analysis by one-way ANOVA with Tukey’s post hoc test. *P*-value indicated. **F** Heat map displaying gene expression of DUSP family members from RNA isolated from *Ptf1a*^*creERT/+*^*KRAS*^*G12D/+*^ and *APK* organoids. Each row indicates a different organoid line. **G** Western blot analysis of phosphorylated (p) ERK and total (t) ERK protein levels in *Ptf1a*^*creERT/+*^*KRAS*^*G12D/+*^ and *APK* organoids. Each lane represents an individual line. **H** Quantification of pERK relative to tERK western blots. Data displayed as mean ± SEM. Statistical analysis by *t*-test with *p*-value indicated.

Since deletion of ATF3 reduced KRAS signaling and transformation in acinar cells (Figs. 1 and 2), but eventually resulted in the enrichment of genes involved in KRAS signaling in organoids (Fig. 3G), we examined mediators of KRAS signaling in vivo. IHC for phosphorylated ERK (p-ERK) 14 days after cerulein treatment showed substantial accumulation in PanINs of *Ptf1a*^*creERT/+*^*KRAS*^*G12D/+*^ mice. P-ERK was minimally reduced in *APK* but showed a marked reduction in *A*^*acinar*^*PK* tissue (Fig. 7A). Since KRAS expression was similar in all lines, we examined RNA-seq data from whole tissue of non-treated mice and identified significantly increased expression of *Dual-specificity phosphatase* (*Dusp5*; Fig. 7B) and *Dusp6* (Fig. 7C) expression in *APK* mice relative to wild type and *Ptf1a*^*creERT/+*^*KRAS*^*G12D/+*^ mice. DUSPS are phosphatases that inhibits MAPK signaling by dephosphorylating ERK [39]. ChIP-seq from a previous study [34] showed ATF3 enrichment at both *Dusp5* and *Dusp6* genes during pancreatic injury (Fig. 7B, C), suggesting ATF3 directly repressed *Dusp5* and *Dusp6* expression. IHC for DUSP6 showed consistently higher accumulation in *A*^*acinar*^*PK* tissue acini (7.3% ± 4.8%; Fig. 7B, C) compared to *APK* (0.6% ± 0.5%) and *Ptf1a*^*creERT/+*^*KRAS*^*G12D/+*^ tissue (0.4% ± 0.2%; Fig. 7B, C), although there was variability in expression, which reflected the extent of acinar tissue remaining following cerulein treatment.

Due to the TME present in the pancreas, we returned to the organoid cultures to assess inhibitors of MAPK signaling. Mapping of the DEGs within the KEGG MAPK signaling pathway (Supplemental Fig. S7) confirmed increased expression of negative regulators of this pathway in *APK* organoids. However, while heat maps showed an increase in *DUSP* expression in all *APK* organoid lines relative to *Ptf1a*^*creERT/+*^*KRAS*^*G12D/+*^ organoids, the *DUSP* isoform increased in each line differed (Fig. 7F). Regardless of this variability, pERK levels in APK organoids were consistently lower relative to *Ptf1a*^*creERT/+*^*KRAS*^*G12D/+*^ organoid cultures (Fig. 7G, H). Together, this data shows the absence of ATF3 diminished KRAS signaling during KRAS^G12D^-mediated transformation, suggesting ATF3 is required for KRAS signaling during early transformation, potentially through the repression of negative regulators of KRAS signaling. These findings are consistent with an intrinsic, epithelial-specific role for ATF3 in promoting KRAS^G12D^-mediate oncogenesis in acinar cells.

## Discussion

Previous work from our laboratory showed global loss of ATF3 restricted oncogenic KRAS^G12D^’s ability to promote PanIN progression [33]. However, ATF3 is expressed in epithelial and non-epithelial cells of neoplastic lesions, and studies in other cancer models suggest cell-specific roles for ATF3 have opposing effects on tumour progression. Therefore, the current study examined an epithelial-intrinsic role for ATF3 in KRAS^G12D^-mediated signalling and determined ATF3 was required for many transcriptomic changes promoted by KRAS^G12D^ in pre-malignant cells. Surprisingly, acinar loss of ATF3 restricted neoplastic lesion progression more than global deletion, suggesting opposing roles for ATF3 in epithelial and non-epithelial compartments of PDAC tumours. In addition, deletion of *Atf3* in acinar cells altered expression of pathways involved in cancer progression through direct and indirect mechanisms, targeting positive and negative regulators of KRAS signaling that lead to decreased signaling through the MAPK pathway.

Our data supports an important role for ATF3 in promoting KRAS^G12D^-mediated transcriptional changes during neoplastic lesion development. Transcriptomic analysis determined that of the 432 genes altered by KRAS^G12D^, 78.2% of these changes were not significant in the absence of ATF3. Published reports show ATF3 promotes progression in other cancers and mediates KRAS signaling directly [40, 41]. For example, analysis of chromatin accessibility in *KPC*, *KC*, and *WT* mice identified ATF3 binding sites as one of the highest enriched motifs in open chromatin regions after KRAS^G12D^ was expressed [41] and ATF3 affects KRAS signaling in breast cancer models by regulating KRAS expression through miRNAs [40]. In Ewing sarcoma, ATF3 positively mediates PI3K/AKT/mTOR signaling [42], which was identified as differentially enriched between and *Ptf1a*^*creERT/+*^*KRAS*^*G12D/+*^ and *APK* organoids. In addition to a role in affecting ADM, our work shows ATF3 regulates gene expression and maintains the neoplastic phenotype in a cell intrinsic fashion. The absence of ATF3 in organoids expressing KRAS^G12D^ resulted in differential expression of >200 genes. Surprisingly, pathways enriched specific in *APK* organoid transcriptomes included KRAS Signaling Up and Epithelial to Mesenchymal Transition (EMT), which was not consistent with the phenotypic differences observed between *APK* and *Ptf1a*^*creERT/+*^*KRAS*^*G12D/+*^ genotypes. Likely, these differences reflect a feedback mechanism that increases expression of upstream mediators due to inhibition of downstream mediators. In support of this compensation mechanism, *APK* organoids show limited evidence of EMT morphologically and phosphorylation of ERK, a downstream mediator of KRAS signaling, is reduced, likely due to increased expression of phosphatases targeting this protein. These results suggest ATF3 mediate KRAS signaling indirectly by affecting the expression and activity of modulating proteins within the RAS signaling cascade.

Further support of this model shows DUSP5 and DUSP6, phosphatases that bind, reduce, and sequester ERK in the nucleus and cytoplasm, respectively [39, 43, 44], are elevated in APK tissue. Acinar-specific loss of ATF3 reduced pERK accumulation and maintained DUSP6 expression, supporting a role for ATF3 in promoting ADM by repressing DUSP6. Preventing this negative regulatory pathway would lead to increased pERK and enhanced KRAS^G12D^-driven tumorigenesis [45]. Decreased DUSP6 correlates to increased migration in PDAC [46] and ATF3 regulates DUSP6 expression in secondary acute myeloid leukemia through direct binding of the *Dusp6* gene [47]. ATF3 overexpression has been linked to altered DUSP expression in other cancer models, including thyroid cancer [48]. Our previous ChIP-seq analysis showed ATF3 enrichment at both *Dusp5* and *Dusp6* genes 4 h after inducing of injury, suggesting these are direct targets of ATF3 transcriptional activity [34]. However, while pERK activity is reduced in all *APK* organoid lines, there is not a consistent increase in *Dusp* gene expression. *Dusp5* and *Dusp6* are elevated in one *APK* line, while *Dusp7* and *Dusp10* are elevated in other *APK* lines, derived from separate animals. The increase in the different phosphatases suggests a less defined effect upon losing ATF3 function and could account for the variability observed between mice and organoid lines.

Other pathways linked to MAPK signaling and altered in the absence of ATF3 may be affected in the same fashion with differences between early (i.e., in acini) and late (i.e., organoids) stages of ADM. ROS signaling was positively enriched in the presence of ATF3, and GSEA showed significant enrichment for PI3K/AKT/MTOR signaling and EF2 targets specifically in *Ptf1a*^*creERT/+*^*KRAS*^*G12D/+*^ acini, indicating ATF3 affects several pathways previously linked to PDAC. However, the PI3K/AKT/MTOR pathway was enriched in *APK* organoids. Like KRAS/MAPK signaling, this pathway is regulated, in part, by downstream, post-translational phosphorylation. Therefore, ATF3 likely contributes to PDAC malignancy by maintaining several signaling pathways, including MAPK and PI3K/AKT/MTOR, through repression of genes encoding phosphatases that target these pathways.

Loss of ATF3 resulted in reduced survival of epithelial cells in organoid cultures (Fig. 4) and GSE analysis showed enrichment of the apoptotic gene signature when ATF3 is lost. There is a well-established body of literature regarding ATF3’s role in cell survival [49]. ATF3 directly regulates apoptosis through targeting apoptotic genes. ATF3 binds to the promoter of *Death receptor 5*, a member of the death-inducing signal complex, to upregulate transcription and promote apoptosis [50]. In models of ischemia-reperfusion injury, ATF3 loss increases apoptosis in cardiac macrophages ex vivo and in vivo [51]. In human colorectal cancer cells, ATF3 overexpression increased expression of the pro-apoptotic marker cleaved PARP (Poly(ADP-ribose) Polymerase [52]) and ATF3 directly binds to the promoter and represses transcription of the pro-survival gene Bcl-xL [50]. *APK* organoids show increased cell death, but only modest increases in the pro-apoptotic cleaved caspase 3 and decreases Bcl-xL. While this supports a role for ATF3 in regulating apoptosis in epithelial cells during PDAC initiation, likely other forms of cell death are involved. ATF3 has been linked to other forms of programmed cell death, such as ferroptosis [53] and non-programmed necroptosis [29]. Therefore, future experiments will address ATF3 role in regulating these forms of cell death during PDAC initiation.

Importantly, our work supports conflicting roles for ATF3 between epithelial and non-epithelial compartments of developing neoplastic lesions. In many cancers, including pancreatic cancer, cells of the tumour microenvironment affect progression of cancer pathology and scRNA-seq identified ATF3 in subsets of fibroblasts and immune cells for both murine and human models of PDAC [33]. We identified differences in the degree of neoplasia when ATF3 was targeted globally (*APK*) or specifically in acinar cells (*A*^*acinar*^*PK*). The differences between *APK* and *A*^*acinar*^*PK* mice supports ATF3 roles in non-epithelial cells of PDAC tumours. Results showing *APK* mice have more overt lesions compared to *A*^*acinar*^*PK* mice suggest ATF3 limits progression through non-epithelial cell intrinsic mechanisms. While pro- and anti-oncogenic properties for ATF3 have been identified in other cancers, only studies in breast cancer revealed opposing roles for ATF3 in the same tumour. ATF3 restricts tumour growth at the primary tumour site but promoted establishment of cancer cells at metastatic sites [54]. While opposing roles were not based on cell type expression, loss of ATF3 in epithelial cells likely had indirect effects on cell composition of the tumour microenvironment. In Ewing sarcoma, ATF3 promotes expression of chemokines that stimulate an M2 macrophage phenotype [42]. Previously, we showed loss of ATF3 decreases macrophage infiltration following KRAS^G12D^-mediated tissue transformation [33]. Preliminary data from our lab indicates acinar-specific loss of ATF3 similarly leads to reductions in macrophage infiltration (data not shown), indicating a cell extrinsic role for epithelial ATF3 during tissue transformation. Further studies are required to determine whether ATF3’s role in macrophage biology during oncogenesis is macrophage intrinsic, epithelial extrinsic, or both. In melanoma, breast, and lung cancer, ATF3 is correlated to an anti-tumour effect in fibroblasts through direct and indirect effects on cancer cells. In skin cancer models, ATF3 negatively regulates CAF activation [55], and in melanoma models, ATF3 overexpression in fibroblasts has an anti-tumour effect on growth and migration [56].

Given ATF3’s expression is limited to the pathological state and not expressed in healthy pancreatic tissue, it could be considered an attractive therapeutic target. Expression of ATF3 in epithelial cells appears to maintain early stages of PDAC progression and we [33] and others [57] previously showed ATF3 is expressed in epithelial, fibroblast, and immune cells of established tumours [33]. However, since ATF3 is an adaptive response gene, its regulation of target genes likely changes in response to shifting stimuli. Similar to TGFβ signaling, ATF3’s role and regulation of target genes likely changes following tumour initiation, growth, and metastasis. Therefore, further analysis in models and patients will be required to evaluate ATF3’s role in later stages of PDAC and in the TME. In addition, there are currently no ATF3 pharmacological inhibitors, and caution is necessary given the opposing roles of ATF3 in PDAC. However, inhibitors of PERK signaling, which ATF3 helps mediate, do have anti-cancer activities[58, 59] and could be evaluated for ATF3 modulation.

Overall, this assessment underscores the complexity of ATF3 within pancreatic cancer and highlights the importance of context in determining ATF3’s role in carcinogenesis. Given the differences observed between *APK* and *A*^*acinar*^*PK* mice in this study, further work is required to identify cell-specific roles for ATF3 in the developing tumour microenvironment during PDAC initiation and progression. Given the highly complex interactions of tumour cells and the microenvironment, understanding the role for ATF3 in these cell types could identify targets for increasing efficacy in PDAC therapy.

## Methods

### Mouse models

Two- to four-month-old male and female mice were used in all studies. Mice were established in a C57/Bl6 background and provided food and water ad libitum. Matings and experimental procedures were approved by the Animal Care Committee at the University of Western Ontario (animal protocols #2020-157; #2020-158). To induce cre-mediated recombination, mice received 5 mg of tamoxifen (Sigma Aldrich, cat. #10540-29-1, St. Louis, Missouri, USA) once a day for 5 days via oral gavage.

Mice allowing tamoxifen-inducible expression of *KRAS*^*G12D*^ involved a targeted insertion of creERT into the *Ptf1a* genes (*Ptf1a*^*creERT*^) and *KRAS*^*G12D*^ targeted to the *Kras* gene preceded by a *loxP-stop-loxP* (*LSL*) cassette (*Kras*^*G12D/+*^). These mice were previously described (*Ptf1a*^*+/creERT*^*Kras*^*G12D/+*^; [60] and were mated to mice harbouring a germline deletion of *Atf3* (*Atf3*^*−/−*^; [61] to generate *Atf3*^*−/−*^*Ptf1a*^*+/creERT*^*Kras*^*-G12D/+*^ mice (*APK*; [33] or mice containing lox-P sites flanking the translational start site of the *Atf3* gene [62] to allow acinar specific-deletion of *Atf3* (*Atf3*^*fl/fl*^*Ptf1a*^*+/creERT*^*Kras*^*G12D/+*^; *A*^*acinar*^*PK*; Supplemental Fig. S5A). Genotypes were confirmed before and after experimentation using primers as described previously [34].

### Acinar cell isolation and culture

For acinar cultures, mice were euthanized 22 days after initial TX gavage and pancreatic tissue dissected away from fat and other internal organs. Acinar cells were isolated as described previously [63]. Portions from the head and tail of the pancreas were fixed in formalin and the rest used for acinar isolation. Pancreata were washed with PBS and incubated with 1 mg/ml collagenase type IV (Worthington, cat #4188) at 37 °C for 15 min. Tissue was mechanically digested in HEPES buffer and cell suspension filtered through a 70 µm nylon mesh filter (Fisher, cat. #07-201-431). Filtered cells were centrifuged for 1 min at 200 × *g*, then resuspended in HEPES. 50,000 cells/well were plated on a 1:1 mixture of rat tail collagen (Corning, 354236) and DMEM/F12 (Gibco, cat #11330-032, Grand Island, New York, USA). Cells were cultured with DMEM/F12 supplemented with 10% FBS (Gibco, cat #12483020), 1% penicillin-streptomycin (Fisher, cat #SV30010, Fair Lawn, New Jersey, USA), 0.04 mg/mL soybean trypsin inhibitor (Gibco, cat #17075-029), and 1 µg/mL dexamethasone.

### Cerulein-induced pancreatitis (CIP) and organoid cultures

To induce acute pancreatic injury, mice received 8 hourly injections of cerulein (50 μg/kg of body weight; Med Chem Expression, cat.#HY-A0190/CS-5876, Monmouth Junction, New Jersey, USA) 15 and 17 days after initial TX gavage. Mice were euthanized two weeks after final CIP treatment. Tissue was used for histological analysis (see below) or to establish organoid cultures. Pancreata was digested in 1 mg/ml of collagenase/dispase for 20 min at 37 °C. Digested tissue was washed with DMEM/F12 containing 10 mM HEPES, 1% glutamax, 1% PenStrep and 100 μg/mL primocin and centrifuged at 300 × *g* for 5 min. The resulting slurry was incubated in StemPro Accutase (Gibco, cat #A11105-01) for 45 min at 37 °C, then filtered through a 70 µm nylon mesh filter. Filtered cells were resuspended in feeding media (Huch et al., [64]) plus 5% Matrigel and seeded on 100% Matrigel (Corning, cat #356230, Bedford, Massachusetts, USA). Once organoid lines were established, organoids were reseeded into 100% Matrigel domes unless noted otherwise.

### Organoid Imaging

Organoids were seeded at a density of 6000 cells/well for organoid inclusion and organotypic experiments [37, 38]. Experiments were repeated with at least three separate organoid lines derived from different mice for each genotype. The Incucyte S3 Live Cell Analysis Instrument (Sartorius, Ann Arbor, Michigan, USA) was used for visualization and live imaging. Movies were generated using Incucyte S3 software. To generate movies for analysis, images were taken every 4 h for organotypic experiments and every 6 h for inclusion experiments. For propidium iodine analysis, at Day 7, cultures were incubated in 20 µg/mL Propidium Iodide for 1 h at 37 °C and visualized with a Leica Microscope DM5500B DFC365 FX camera with LAS V4.4 software. Images were processed and differences calculated by ImageJ.

### Histological analysis

For histological analysis, the head and tail of the pancreas were fixed in 4% formalin at 4 °C for 72 h. Tissues were washed 3× with PBS, then embedded in paraffin and sectioned to 5 µm. Paraffin sections were stained with H&E to assess general tissue morphology. H&E-stained sections were submitted to a blinded clinical pathologist for classification of lesion severity. Nine to ten animals per genotype were characterized by pathologist assessment.

For IHC, paraffin sections were rehydrated and antigen retrieval performed through heat-induced epitope retrieval in citrate buffer. IHC staining was performed with the VectaStain ABC kit (Vector Laboratories, cat. #PK-4001, Brockville, Ontario, Canada) and ImmPACT DAB peroxidase (HRP) substrate (Vector Laboratories, cat. #SK4105) following kit instructions. Following permeabilization with 0.2% Triton-X in PBS, sections were blocked in 5% sheep serum in PBS for one hour at room temperature. Slides were incubated overnight at 4 °C with primary antibodies diluted in blocking solution specific for amylase (1:2000; Abcam; cat. #ab21156, Toronto, Ontario, Canada), CK19 (1:1000; Abcam, cat. #ab52625), p-ERK (1:50; Cell Signaling, cat. #9101, Danvers, Massachusetts, USA), and DUSP6 (1:100; Abcam, cat. #ab76310). Following incubation, sections were rinsed 3× with PBS, then incubated with a biotinylated secondary antibody (1:400 diluted in blocking solution) specific for rabbit IgG for 30 min at room temperature. Following a second set of rinses in PBS, tissue sections were incubated 30 min at room temperature with AB reagent. Visualization of the target antigen was achieved using DAB peroxidase. Slides were counterstained with hematoxylin (BioCare Medical, ref #CATH-M, Pacheco, California, USA), mounted with Permount (Fisher cat #SP15-100), and imaged using a Leica Microscope DM5500B DFC365 FX camera with LAS V4.4 software.

### Protein isolation and immunoblots

Tissue from the middle of the pancreas was flash frozen in liquid nitrogen for protein isolation or organoids were recovered from Matrigel domes using cell recovery solution (Corning, cat #354253) as described previously [64]. Protein was isolated and western blot analysis performed as described [65]. Primary antibodies for tERK (1:500, Cell Signaling, cat. #9102S), ATF3 (1:1000; Cell Signaling, cat #33593S), Bcl-xL (1:1000; Abcam cat #ab32370), cleaved caspase 3 (1:500; Cell Signaling, cat #9661S), E-cadherin (BD Biosciences cat #610182), N-cadherin (BD Biosciences cat #610921), and GAPDH (New England Biolabs cat #3108S) and secondary antibodies (1:10,000; Cell Signaling, cat #7074S) were diluted in 5% BSA in TBST. Blots were developed with Clarity Western Blot ECL Kit (BioRad, cat. #1705061, Mississauga, Ontario, Canada) and visualized using an Invitrogen iBright1500 system. Complete Western blots can be found in Supplementary Materials. In some cases, blots were divided for detection of different sized proteins.

### RNA isolation, sequencing, and bioinformatics

For transcriptomic analysis, acinar cells were isolated from mice 22-days after tamoxifen gavage as described above or immediately after acinar cell isolation using Trizol (ThermoFisher Scientific, 15596018) and the PureLink RNA mini kit (ThermoFisher Scientific, 12183018A). For differential expression in organoids, RNA was isolated seven days after organoid plating. Organoids were recovered from Matrigel domes using cell recovery solution (Corning, cat #354253) as described previously [64]. Following cell recovery, RNA was isolated using RNeasy Mini Kit (Qiagen, cat #74104, Hilden, Germany) following manufactures instructions. RNA quality was confirmed using an Agilent 2100 Bioanalyzer and sequenced to a depth of 50 million reads/sample using the Illumina NextSeq High Output 75 cycle (acini RNA-seq) or 150 cycle (tissue and organoid RNA-seq) kits.

Sequence quality was assessed using FastQC and reads mapped to the mm10 genome using STAR [66], counted using the featureCounts option in the Subread aligner [67], and analyzed for differential expression using DESeq2 package [68] in RStudio. Batch effect correction and data normalization was performed through built-in DESeq2 features. KEGG and GO pathway analyses were performed using clusterProfiler R package [69], Gene Set Enrichment Analysis (GSEA) was performed using fgsea R package. Gene sets used for enrichment analysis and visualization were obtained from MSigDB [70, 71]. Heatmaps were generated using pheatmap R package [72] and PCAtools [73] and enrichplot [74] were used for generating PCA plots and dotplots.

### Sample size and statistical analysis

All *n* values presented represent biological replicates. Organoid lines were derived from separate animals and data points provided throughout represent individual lines. The only exceptions to this are when organoid size and lesion size are determined over a large number of organoids. In these instances, data was pooled from three lines for each genotype. In every case, experiments were repeated two or three times (i.e., separate days and separate organoid lines/mice). For quantification of IHC and propidium iodine images, files were uploaded to ImageJ by a blinded lab member and quantified. The numbers of lines generated and the number of mice used to achieve significance were based on previous studies in our and other laboratories. No power analysis or randomization was performed. Data was analyzed for significance by an unpaired, two-tailed *t*-test unless indicated otherwise, or by 1-way analysis of variance (ANOVA) with Tukey post hoc test. Values are depicted as means ± standard error of the mean. Significance is considered *P* < 0.05.

## Supplementary information

Wild type acinar cells in collagen

Acinar cells from APK mice grown in collagen

Acinar cells from Ptf1acreERT/+-KRASG12D mice grown in collagen

Ptf1acre/ERT/+-KRASG12D organoids grown in matrigel

APK organoids grown in matrigel

Ptf1acre/ERT/+-KRASG12D organoids grown on matrigel

APK organoids grown on matrigel

Supplementart Table S1. List of DEGs between Wild Type and Ptf1acre/+KrasG12D acinar cells

Supplementary Table S2. DEGs between Ptf1a+/creERTKRASG12D and APK acini

Suplementary Table S3. DEGs between Ptf1a+/creERTKRASG12D and APK Organoids

Supplementary figures

Complete Western blots

Dataset 1

## Data Availability

RNA-seq data from acinar cells and organoids is available at the NIH GEO database (accession #GSE2699380). RNA-seq from pancreatic tissue was initially published in[75] is available from the NIH GEO database (accession #GSE252884).
