# Supplementary material for: Acinar-specific loss of activating transcription factor 3 restricts KRASG12D mediated transcriptional changes and PanIN progression
Source: Cell Death Discov. 2025 Nov 6;11:503. doi: 10.1038/s41420-025-02777-2 (PMC12592554; doi:10.1038/s41420-025-02777-2)
Supplement: Supplementary file 8 — Supplementart Table S1. List of DEGs between Wild Type and Ptf1acre/+KrasG12D acinar cells [file 41420_2025_2777_MOESM8_ESM.pdf]

Supplemental Table S1. List of DEGs between Wild Type and Ptf1a$^{cre/+}$Kras^{G12D} acinar cells

Higher expression in *Ptf1a$^{cre/+}$ Kras^{G12D}*				
Symbol	Ptf1a$^{cre/+}$Kras^{G12D} vs Wild Type		*APK* vs Wild Type	
	log2 Fold Change	pvalue	log2 Fold Change	pvalue
Batf3	18.75	2.82E-06	15.58	0.000116
Snord104	15.81	9.16E-05	17.53	1.25E-05
Spink4	15.79	9.30E-05	18.49	3.24E-06
Snhg9	15.79	9.37E-05	18.08	6.00E-06
Mir8100	15.24	0.0001691	16.99	2.32E-05
Guca1b	15.24	0.0001691	16.99	2.32E-05
Xist	10.60	0.0021131	1.11	0.754145
Cfd	8.00	0.0376387	-2.91	0.46311
Gkn3	5.47	0.0005079	3.27	0.044506
Sox15	5.00	0.0107983	3.70	0.064956
Ccno	4.98	0.0050904	5.10	0.003793
Greb1	4.61	0.0078139	4.73	0.006072
6430573P05Rik	4.54	0.0240016	4.50	0.024277
Pgc	4.53	0.0020492	4.03	0.006189
Gcnt3	4.30	0.0339451	5.21	0.008472
Ccr6	4.21	0.0461566	1.85	0.394057
Edil3	4.21	0.0063279	0.93	0.576546
Myl10	4.14	0.0083058	3.19	0.044118
Kcnh5	4.12	0.0321034	2.63	0.178977
Gm9136	4.09	0.0453971	2.73	0.189373
Fkbp1b	3.92	0.0315296	-0.38	0.848033
Thbs4	3.89	0.0089267	2.86	0.057564
Tnc	3.66	1.97E-05	2.75	0.001434
Xcr1	3.65	0.044867	-1.30	0.534472
Phlda2	3.57	0.0227391	1.96	0.220418
Ackr2	3.39	0.0496817	2.34	0.179304
Trarg1	3.36	0.0190205	-0.21	0.891413

Lower expression in *Ptf1a$^{cre/+}$ Kras^{G12D}*				
Symbol	Ptf1a$^{cre/+}$Kras^{G12D} vs Wild Type		*APK* vs Wild Type	
	log2 Fold Change	pvalue	log2 Fold Change	pvalue
B4galnt2	-17.66	1.13E-05	-17.75	1.02E-05
Krt1	-17.66	1.13E-05	-17.75	1.02E-05
Mrgprh	-17.66	1.13E-05	-17.75	1.02E-05
Prr16	-17.66	1.13E-05	-17.75	1.02E-05
Trpc3	-17.66	1.13E-05	-17.75	1.02E-05
Igkv17-127	-17.66	1.13E-05	-17.75	1.02E-05
Igkv4-57-1	-17.66	1.13E-05	-17.75	1.02E-05
Eomes	-17.66	1.13E-05	-17.75	1.02E-05
Nphp4	-16.54	3.97E-05	1.84	0.64172
Gm18343	-16.37	4.75E-05	1.84	0.64076
Acat3	-16.09	6.42E-05	1.85	0.639
Gm14425	-16.09	6.42E-05	1.85	0.639
Gm16090	-15.95	7.52E-05	1.86	0.63783
Id4	-4.74	0.02566	-1.99	0.29067
Tgtp2	-4.62	0.00734	-1.37	0.29891
Stap1	-4.59	0.02922	-1.55	0.398
H2-Q5	-4.57	0.00138	-2.23	0.04672
Pcdhb18	-4.53	0.03923	-3.47	0.10554
D030047H15Rik	-4.45	0.02962	-1.82	0.31284
Nxpe2	-4.42	0.00311	-3.40	0.01245
Prox2	-4.37	0.01595	0.02	0.98877
Angptl7	-4.35	0.00628	-2.39	0.07746
B230216N24Rik	-4.34	0.04695	-3.06	0.14691
Gm17068	-4.33	0.04762	-2.27	0.25999
Wdr54	-4.30	0.0151	-0.03	0.98338
Rpl3	-4.26	1.46E-05	-4.31	9.94E-06
Ifi206	-4.25	0.0457	-1.10	0.55377

Reg3g	3.14	7.74E-43	2.65	5.98E-31	Dbpht2	-4.21	0.01305	-1.25	0.36989
Cnr2	3.01	0.0414933	0.51	0.742232	Cfap251	-4.20	0.02842	-0.21	0.89331
H3c8	2.98	0.0138349	1.91	0.120431	Cep112	-4.17	0.00709	-0.09	0.93475
Ckap2l	2.97	0.0403253	2.60	0.07288	Slc4a1	-4.16	0.00493	-1.02	0.40037
Reg3a	2.90	1.01E-05	2.22	0.000703	AA465934	-4.12	0.01105	-0.58	0.62312
Atp1a3	2.85	0.0168003	1.95	0.105241	Phf11c	-4.10	0.01523	-2.93	0.05238
Gm2663	2.84	0.0014685	2.05	0.022609	Sh3bgr	-4.06	0.02895	0.09	0.9538
Abcc6	2.83	0.0059322	1.68	0.108517	Afap1l2	-4.00	0.00048	-0.40	0.61699
Noxa1	2.81	1.50E-12	2.56	1.11E-10	Hdc	-3.92	0.00381	-3.38	0.0087
H3c3	2.80	0.0365933	1.60	0.234569	Qprt	-3.91	0.03541	-2.23	0.16159
Reg3b	2.78	4.68E-08	2.28	7.65E-06	Lrrc18	-3.88	0.03976	-3.69	0.04894
Was	2.77	0.0371759	-1.24	0.403533	Armh4	-3.87	0.04169	0.39	0.80295
Odad4	2.70	0.0138213	1.73	0.120519	9530059O14Rik	-3.85	0.01324	-2.45	0.06832
Pxmp2	2.67	0.047311	2.13	0.11386	Ptprz1	-3.84	0.03359	-1.01	0.50598
Rpl28	2.66	0.0430058	1.32	0.32303	Cdh2	-3.81	0.04767	0.67	0.6676
Calr3	2.65	0.0363235	0.41	0.75606	Nacad	-3.77	0.04669	-1.82	0.25638
Cfi	2.62	0.002166	2.26	0.008288	AW551984	-3.71	0.01901	-2.05	0.14088
Rad51b	2.56	0.0139294	2.22	0.033317	Stat4	-3.70	0.02433	-1.51	0.28896
H4c6	2.36	0.0008502	0.41	0.570156	AI662270	-3.69	0.00644	-1.27	0.23411
Wfdc17	2.36	0.0130902	1.62	0.091536	Slc24a2	-3.60	0.00389	-1.67	0.10523
Chil6	2.34	0.0493037	2.85	0.0147	Zfp951	-3.59	0.02866	-0.39	0.771
Etv5	2.34	3.64E-10	2.81	2.52E-14	Gm4951	-3.52	0.03075	0.10	0.94334
Gm11405	2.33	0.0488879	0.35	0.774252	Folh1	-3.51	0.03512	-2.85	0.07482
Alox8	2.31	0.0108065	0.79	0.40113	Fbxw10	-3.51	0.02321	-0.40	0.74522
Bambi	2.27	0.0237572	1.08	0.288652	Epha5	-3.49	0.0258	-2.36	0.09116
Grik4	2.21	0.0358476	0.55	0.609268	Dkkl1	-3.40	0.04203	0.39	0.76661
Smim22	2.19	0.0377022	1.33	0.210697	Aox3	-3.37	0.03525	-2.29	0.13759
Proc	2.12	0.0423472	0.33	0.762902	Gm16023	-3.36	0.01006	-0.58	0.56214
Dlk1	2.08	0.0337239	2.20	0.023598	Neb	-3.36	0.04827	-1.50	0.32196
1810062G17Rik	2.05	0.0044541	0.94	0.201166	Kcnma1	-3.33	0.02615	-1.45	0.25202
H4c12	2.02	0.0024279	0.65	0.342784	Scube3	-3.29	0.03889	-1.47	0.26984
Ifi27l2b	2.02	0.0115616	1.58	0.049484	Map10	-3.25	0.04812	-1.24	0.37468
Rtn2	1.99	0.0438871	-0.36	0.73191	Tnfrsf22	-3.23	0.04914	-1.12	0.40903

Agr2	1.97	0.0004861	1.86	0.000923	Capsl	-3.21	0.04225	-1.41	0.28533
Smim27	1.95	0.0027557	1.35	0.038535	Pde10a	-3.17	0.01467	0.23	0.81281
Gm14706	1.93	0.0373461	0.52	0.584096	Bbs12	-3.07	0.01392	-1.62	0.13575
Phldb3	1.91	0.026516	1.55	0.071223	Itga8	-3.06	0.02472	-0.30	0.78173
Chadl	1.89	0.0264466	0.31	0.722086	Amy2a5	-3.04	0.03541	-1.52	0.23315
Egr2	1.87	0.0060086	0.10	0.891374	Ltbp2	-3.02	0.03901	-0.88	0.48444
H2ac10	1.85	0.0369579	1.19	0.183418	Kel	-2.91	0.04224	-4.63	0.00306
Msr1	1.85	0.0409965	1.04	0.253367	Igkv1-117	-2.89	0.02346	-1.70	0.13968
Tspan1	1.84	5.78E-05	0.78	0.091861	Cela3a	-2.85	1.42E-09	-3.09	3.11E-11
Umod	1.78	0.0009274	1.19	0.027635	B3galt2	-2.85	0.00089	-0.85	0.24499
Nt5dc2	1.77	0.0170572	0.76	0.311937	Itk	-2.82	0.02246	-1.70	0.11966
Prdm5	1.75	0.0235917	0.06	0.94218	Gpr22	-2.78	0.0385	0.49	0.63721
Cnr1	1.75	0.0011971	1.74	0.0012	Loxl4	-2.74	0.01335	-1.58	0.10339
Taf1a	1.75	0.0373539	1.41	0.093109	Rimbp2	-2.70	0.04623	-0.11	0.92332
Gm22442	1.75	0.0011916	0.73	0.183522	Xaf1	-2.68	0.00298	-0.52	0.49158
Slc15a2	1.75	0.0003093	0.77	0.115309	Pla2r1	-2.66	0.04187	-0.99	0.37985
Gpt	1.71	1.52E-08	1.49	8.75E-07	Slc16a11	-2.65	0.03929	-0.51	0.64071
H1f5	1.66	0.0437779	-0.05	0.952813	Gm15787	-2.64	0.04092	-1.56	0.17835
F2	1.66	0.0001968	1.60	0.000315	Crp	-2.64	0.0189	0.50	0.59934
Trmt61b	1.65	0.0228057	1.30	0.071808	Slc7a14	-2.62	0.03759	-0.41	0.70554
Prkar1b	1.63	0.0449598	1.36	0.093885	Plet1	-2.58	0.03498	-0.22	0.82849
H4c2	1.62	0.0329967	0.56	0.464851	Lvrn	-2.55	0.0046	0.01	0.98465
Esr1	1.62	0.0085471	2.08	0.000586	Il18r1	-2.54	0.01393	-1.83	0.05291
Mpg	1.60	0.0004138	0.88	0.05277	Serpina6	-2.52	0.03373	-3.16	0.009
Cdk2ap1	1.59	0.0257826	1.18	0.097399	Hoxa5	-2.50	0.04725	-0.85	0.42767
Lrg1	1.59	6.33E-05	1.66	2.30E-05	Rab37	-2.48	0.04913	-1.04	0.35916
C2cd4b	1.58	0.0164533	0.48	0.468387	Iyd	-2.45	3.73E-06	-3.30	1.08E-09
C4b	1.54	1.28E-07	1.59	5.02E-08	Irag1	-2.35	0.00889	-0.74	0.34914
Dok1	1.50	0.011448	1.31	0.025916	Cdh13	-2.35	0.03445	0.47	0.61727
Reg2	1.50	1.37E-05	1.39	5.58E-05	Ust	-2.30	0.02876	-1.17	0.23419
Ttr	1.49	1.13E-06	0.81	0.008231	Tbx2	-2.29	0.04776	-0.92	0.37493
Cd59a	1.48	0.0007111	0.82	0.062537	Filip1	-2.29	0.01697	-1.46	0.09946
Trip6	1.46	0.0043426	0.85	0.100684	Frzb	-2.28	0.0272	-0.75	0.4076

Slc6a4	1.45	4.03E-05	1.53	1.26E-05	Pygo1	-2.27	0.02998	-1.09	0.25534
Atf3	1.43	0.0001429	-0.36	0.334521	Sema3d	-2.27	0.01356	0.69	0.37505
H4c16	1.42	0.0086241	0.64	0.235599	Plxna4	-2.26	0.00021	-0.44	0.42912
Rab17	1.42	0.0399774	0.61	0.381877	Zfp185	-2.22	0.02625	0.06	0.9417
H2bc13	1.39	0.0323751	0.05	0.941494	Pak3	-2.21	0.044	-1.30	0.20076
Fetub	1.38	0.0422972	1.53	0.02478	Rbp7	-2.19	0.04557	-0.47	0.63031
Gpank1	1.37	0.0056396	0.85	0.088906	Btnl9	-2.18	0.00085	-0.85	0.15043
H4c11	1.36	0.0411665	0.43	0.52729	Pcdhgb4	-2.17	0.04397	-1.26	0.20154
Pafah1b3	1.36	0.0447601	0.37	0.585796	Slc2a2	-2.15	0.00458	-0.40	0.56433
Ppp1r35	1.35	0.0084961	0.66	0.205001	Ttn	-2.15	0.00319	-0.09	0.89326
Socs3	1.35	8.68E-05	0.72	0.036309	Adam33	-2.14	0.01622	-0.98	0.2403
Cyp4f16	1.33	0.0445748	0.11	0.868152	Cep170	-2.10	0.00028	-0.78	0.14691
Bex2	1.32	0.0011643	0.98	0.016158	Sh3tc2	-2.10	0.01635	0.04	0.95603
Megf6	1.32	0.0497119	1.30	0.05261	Mab21l3	-2.08	0.00942	-1.91	0.01309
Lipt2	1.31	0.0427727	0.75	0.247342	Sphkap	-2.07	0.00592	0.09	0.89301
Glns-ps1	1.31	0.0306479	1.20	0.045361	Xrcc3	-2.06	0.02467	-0.48	0.55096
Endog	1.30	0.0172997	0.64	0.247141	Myh11	-2.04	0.00585	-1.21	0.07521
Ier2	1.30	1.82E-06	0.42	0.124575	Pcdhgb2	-2.04	0.03183	-1.47	0.10113
Car11	1.29	0.0357703	0.85	0.165341	Kcnh1	-2.03	0.00865	-1.79	0.01765
Prxl2b	1.28	0.0382892	-0.32	0.617935	Sgpp2	-2.02	0.04699	0.19	0.82986
Thap3	1.28	0.0272272	0.48	0.409661	Aqp5	-2.01	0.03078	-1.18	0.17601
Dhtkd1	1.27	0.0085354	0.34	0.481423	Cycs	-1.99	0.04301	-0.07	0.93392
Bloc1s2	1.26	0.047381	1.03	0.103191	Exoc3l	-1.98	0.03565	-0.84	0.32642
Ndrg2	1.26	0.0031541	0.78	0.067019	Pkia	-1.95	0.0138	-0.92	0.21691
Eepd1	1.25	0.0006849	0.88	0.017162	Gm28071	-1.90	0.04848	-0.28	0.73916
Cyb5d1	1.25	0.0491732	0.70	0.267586	Kif5c	-1.89	1.62E-05	-1.49	0.00047
Ier3	1.24	0.0050137	0.70	0.112447	Wt1	-1.89	0.00106	-1.53	0.00511
H3c7	1.24	0.0181245	0.95	0.06928	Cntrob	-1.89	0.04125	-0.14	0.86581
Polr2j	1.23	0.0221479	0.44	0.415292	Syt7	-1.88	0.00481	-0.47	0.44941
Pih1d1	1.23	0.0015083	0.63	0.10627	Ifi202b	-1.87	0.0133	-0.47	0.49546
Rundc1	1.22	1.01E-05	1.15	3.14E-05	Has1	-1.85	0.0181	-0.90	0.21186
Cyth4	1.22	0.0326491	0.13	0.817222	Gal	-1.85	8.57E-09	-2.71	3.86E-17
Chchd5	1.21	0.0049452	0.41	0.350921	Spry4	-1.85	0.00232	-0.68	0.2226

Coa4	1.21	0.0329942	0.38	0.505953
Scamp5	1.20	0.0251823	0.60	0.261767
Dusp23	1.19	0.0386536	-0.05	0.929645
Slc25a35	1.19	1.05E-06	1.38	1.09E-08
Soat2	1.18	0.0211313	1.09	0.031722
Snord17	1.17	0.0082949	1.28	0.003608
Pet100	1.17	0.0134435	0.38	0.427816
Fos	1.16	2.11E-05	0.45	0.097756
Ccl9	1.16	0.0342102	0.67	0.221886
Gm6652	1.15	0.0002837	0.75	0.01684
Slc7a7	1.14	0.012444	0.59	0.198084
Junb	1.14	0.0001783	0.55	0.073411
Pam16l	1.14	0.0183662	0.49	0.312652
Ngef	1.12	0.0155089	0.83	0.071218
Pigf	1.12	0.0108036	0.41	0.357226
Cyba	1.11	0.0091953	0.43	0.312282
Cdk5rap1	1.11	0.0376758	0.63	0.237899
Slc44a4	1.10	0.0132261	0.61	0.168916
Pgp	1.08	0.0179447	0.76	0.094789
Dph1	1.07	0.044056	0.76	0.155338
Maff	1.07	0.0279711	0.76	0.11776
Nt5m	1.06	0.0149666	0.49	0.259884
Adat2	1.05	0.0371599	0.40	0.425036
Rpl35	1.05	5.95E-05	0.49	0.059286
St6gal1	1.05	0.0081143	0.73	0.062876
Rpl36-ps12	1.04	0.0083168	0.10	0.81005
H2ac20	1.04	0.0219348	0.90	0.044959
Cldn7	1.03	0.0001057	0.55	0.039521
Clp1	1.02	0.0299002	0.43	0.364435
Ilvbl	1.01	0.0032491	0.35	0.306591
Myo15b	1.00	0.0002898	0.87	0.001723
Tspo	1.00	0.0026915	0.47	0.156782
Ksr1	-1.84	0.00486	-0.69	0.25358
Cx3cr1	-1.83	0.01013	0.29	0.63995
Cwc27	-1.83	0.01421	-0.18	0.79221
Meg3	-1.82	0.0165	-0.20	0.77193
Pear1	-1.78	0.00302	-0.50	0.35697
Trib2	-1.77	0.0104	-0.96	0.14253
BC034090	-1.76	5.12E-06	-1.89	8.67E-07
Jam2	-1.75	5.55E-05	-0.84	0.0378
Ccdc162	-1.73	0.04887	-1.40	0.09685
Flrt1	-1.73	0.01778	-0.68	0.32507
Tsga10	-1.73	0.01562	-1.23	0.07066
Emb	-1.71	0.01217	-0.54	0.37862
Ccdc82	-1.71	0.03953	-0.97	0.20961
Zfp945	-1.70	0.00328	-0.08	0.87868
Plag1	-1.69	0.04487	-0.71	0.38204
Cacna2d1	-1.69	0.01187	0.14	0.81464
Gbp2b	-1.68	0.0249	-0.45	0.53189
Nhsl2	-1.68	0.01768	-0.09	0.88774
Lmo2	-1.67	0.0118	-0.52	0.40043
Chaserr	-1.64	0.03117	-0.01	0.9829
Cyyr1	-1.64	0.00086	-0.35	0.44896
Plekhg1	-1.62	0.00931	-0.41	0.47734
Lox	-1.62	0.0034	-0.31	0.54578
Tmtc1	-1.61	0.02002	-0.46	0.47607
Pkn3	-1.60	0.02222	-0.63	0.3301
Cntln	-1.58	0.04505	-0.07	0.9187
Kcnq1ot1	-1.58	1.35E-05	-0.65	0.06901
Igtp	-1.58	0.03006	-1.09	0.12348
Pcdh17	-1.57	0.01544	-0.92	0.13388
Zbtb34	-1.57	0.00094	-0.02	0.97206
Hba-a1	-1.56	0.00157	-0.01	0.97864
Malat1	-1.52	2.88E-07	-0.39	0.18135
She	-1.51	0.01508	-0.91	0.12714

indicates gene expression with a p value >0.05 and <0.1

indicates gene expression with a p value >0.1

Thnsl1	-1.51	0.01441	-0.30	0.60599
Dgke	-1.50	0.0031	-0.63	0.18791
Unc5b	-1.50	0.00082	-1.26	0.00357
Neat1	-1.49	2.31E-05	-1.01	0.00374
Lrif1	-1.48	0.00815	-0.06	0.90216
Nsun6	-1.47	0.04845	0.17	0.80413
Ift81	-1.46	0.04463	-0.34	0.61247
Shank1	-1.46	0.0002	-1.17	0.00237
Piezo2	-1.46	0.04971	-0.84	0.24079
Cobl	-1.45	0.00063	-0.38	0.33931
Notch4	-1.45	0.00697	-0.32	0.53054
Gucy1a1	-1.44	0.02132	-0.92	0.12908
Zeb1	-1.44	0.01463	-1.14	0.04915
Hba-a2	-1.44	0.01006	-0.15	0.7872
Kdr	-1.44	3.32E-06	-0.46	0.1262
Cep290	-1.43	0.01455	0.08	0.88482
Gucy1a2	-1.43	0.04603	-1.13	0.10003
Rnpc3	-1.42	0.00663	-0.20	0.68323
Cdh11	-1.42	0.00197	-0.76	0.08318
Ptprb	-1.41	3.81E-05	-0.67	0.04647
Abca6	-1.41	0.02036	-0.23	0.68217
Dlg2	-1.41	0.03415	-1.01	0.1175
Napb	-1.41	0.04154	-0.88	0.17902
Nxph2	-1.39	0.0111	-1.83	0.00086
Gm16551	-1.39	0.03213	-0.72	0.24888
Lrrn4	-1.38	0.0038	-0.83	0.073
Cd200	-1.37	0.00199	-0.78	0.07021
Sox7	-1.37	0.03563	-0.26	0.67388
Nat8	-1.37	0.0085	-1.54	0.0028
Cnnm1	-1.37	0.04807	-0.18	0.78151
Krt80	-1.35	0.00898	-0.93	0.06013
Hook3	-1.34	0.00029	-0.26	0.464
Mpdz	-1.34	0.00511	-0.53	0.24849

Fhl1	-1.34	0.01174	-0.55	0.28499
Alas2	-1.34	0.03825	0.33	0.58518
Fat4	-1.34	0.01271	-1.03	0.04985
1810064F22Rik	-1.33	0.01933	-0.63	0.25032
Echdc3	-1.33	0.00303	-0.64	0.13823
Enpp4	-1.33	0.01523	-0.26	0.61587
Prkar2b	-1.30	0.02832	-1.11	0.05689
Adgrl4	-1.30	0.00164	-0.97	0.01556
Rgs11	-1.29	5.58E-05	-0.77	0.0128
Syne1	-1.28	0.00228	-0.98	0.01735
4632427E13Rik	-1.28	0.0141	-0.10	0.83972
Gm10334	-1.28	0.02555	-2.19	0.0002
Zfp950	-1.28	0.01144	-1.13	0.02262
Tia1	-1.27	0.00267	-0.67	0.1026
Sdr9c7	-1.27	0.00046	-1.80	6.96E-07
Hmcn1	-1.27	0.02312	-0.16	0.76805
Garre1	-1.26	0.00437	-0.39	0.35433
Dll4	-1.26	0.0393	-0.86	0.14178
Tnfsf10	-1.26	0.00087	-0.42	0.24655
Mapt	-1.25	0.00082	-1.15	0.0016
Rbms3	-1.24	0.00713	0.03	0.95164
Pkd1l2	-1.23	0.0015	-1.11	0.00367
Ppp4r4	-1.22	0.00962	-0.47	0.306
Limch1	-1.22	0.01783	-0.20	0.68458
Parp14	-1.22	0.02237	-0.02	0.96798
Ankrd11	-1.21	0.00168	-0.24	0.52332
Dop1a	-1.21	0.00017	-0.59	0.0565
Iigp1	-1.21	0.03965	-0.24	0.66479
Rasal2	-1.21	0.00659	-0.34	0.42837
Kctd12b	-1.20	0.00979	-0.61	0.17579
Wbp4	-1.19	0.03291	-0.23	0.66358
Hbb-bt	-1.19	0.01512	-0.20	0.67066
Acaca	-1.19	0.0215	-0.05	0.92509

Dock8	-1.19	0.02316	-0.55	0.27707
Gpm6a	-1.18	0.01217	-0.34	0.45217
Mdn1	-1.16	0.00011	-0.32	0.2758
Flt1	-1.15	0.00049	-0.70	0.03069
Gm5771	-1.14	0.00033	-2.64	1.75E-16
Cep162	-1.14	0.03712	-0.30	0.562
Stox2	-1.14	0.0031	-0.40	0.2919
Uvssa	-1.14	0.01172	-0.51	0.23557
Ube2i	-1.14	0.0137	-0.40	0.36496
Slc7a11	-1.14	0.00618	-0.87	0.03381
Pkhd1l1	-1.13	0.00067	-0.61	0.05615
Yae1d1	-1.12	0.0392	-0.34	0.51236
Casp9	-1.12	0.00102	-0.70	0.03668
Pikfyve	-1.12	0.00096	-0.34	0.29321
Phc3	-1.11	0.00013	-0.33	0.2502
Cemip2	-1.11	0.01371	-0.39	0.362
Pdgfd	-1.10	0.04462	0.11	0.83873
Pcdh12	-1.10	0.03293	-0.49	0.32792
Sema5a	-1.10	0.00046	-0.27	0.36812
Arhgap31	-1.09	0.00579	-0.54	0.15774
Usp9x	-1.09	0.00011	-0.12	0.66313
Crybg3	-1.09	0.00252	-0.39	0.25784
Tnrc6b	-1.09	0.00079	-0.33	0.29957
Scg2	-1.08	0.01125	0.01	0.97491
3425401B19Rik	-1.08	0.03542	-0.71	0.15148
Smchd1	-1.07	0.01382	-0.35	0.41643
Dcp2	-1.06	0.00722	-0.07	0.84685
Endod1	-1.06	0.03096	-0.19	0.69386
Myo5a	-1.06	0.01851	-0.48	0.26749
Mtx3	-1.05	0.01733	-0.41	0.33257
Rock2	-1.05	0.0033	-0.32	0.35305
Slc43a3	-1.04	0.00356	0.08	0.80447
Zfp407	-1.04	0.00067	-0.67	0.02338

Heg1	-1.04	0.00488	-0.42	0.25438
Leng8	-1.04	0.00383	-0.50	0.1585
Zbtb20	-1.03	0.0015	0.11	0.72376
Cplane1	-1.03	0.00773	-0.25	0.49573
Klhdc7a	-1.03	0.00072	-0.55	0.06582
Pnisr	-1.03	0.01403	-0.18	0.66428
Irgm2	-1.03	0.0322	-0.17	0.71228
Gja1	-1.02	0.01463	-0.40	0.33088
Mbd5	-1.02	0.01741	-0.17	0.67583
Galnt15	-1.02	0.01416	-0.68	0.09212
Bahcc1	-1.01	0.02155	-0.24	0.57201
Vps13b	-1.01	0.0002	-0.34	0.20723
Cntrl	-1.01	0.03804	0.11	0.81747
Mef2c	-1.01	0.04572	-0.36	0.4548
Ubxn2b	-1.00	0.03237	-0.44	0.33861
Peg3	-1.00	0.01726	0.10	0.79482

indicates gene expression with a p value >0.05 and <0.1

indicates gene expression with a p value >0.1
