# Supplementary material for: Acinar-specific loss of activating transcription factor 3 restricts KRASG12D mediated transcriptional changes and PanIN progression
Source: Cell Death Discov. 2025 Nov 6;11:503. doi: 10.1038/s41420-025-02777-2 (PMC12592554; doi:10.1038/s41420-025-02777-2)
Supplement: Supplementary file 9 — Supplementary Table S2. DEGs between Ptf1a+/creERTKRASG12D and APK acini [file 41420_2025_2777_MOESM9_ESM.pdf]

Supplementary Table S2. DEGs between Ptf1a$^{+/creERT}$KRAS^{G12D} and APK acini

Symbol	log2 Fold Change*	p value	p adj
Nphp4	-18.38	4.58E-06	6.59E-03
Gm18343	-18.22	5.54E-06	7.10E-03
Acat3	-17.95	7.63E-06	8.37E-03
Gm14425	-17.95	7.63E-06	8.37E-03
Gm16090	-17.81	9.00E-06	8.64E-03
Glra4	-4.78	3.06E-02	1.00E+00
5730405O15Rik	-4.70	3.99E-02	1.00E+00
Cdh2	-4.48	1.79E-02	1.00E+00
Prox2	-4.39	1.54E-02	1.00E+00
Slc23a3	-4.37	3.67E-02	1.00E+00
Wdr54	-4.27	1.63E-02	1.00E+00
Armh4	-4.26	2.32E-02	1.00E+00
Atp2b2	-4.24	2.90E-02	1.00E+00
Fam180a	-4.18	1.60E-02	1.00E+00
Sh3bgr	-4.15	2.58E-02	1.00E+00
Hdx	-4.14	4.25E-02	1.00E+00
Cep112	-4.09	8.40E-03	1.00E+00
Has3	-4.04	1.06E-02	1.00E+00
Cfap251	-3.98	3.77E-02	1.00E+00
Ribc1	-3.92	4.72E-02	1.00E+00
Gm16465	-3.89	3.58E-02	1.00E+00
Plk-ps1	-3.85	4.80E-02	1.00E+00
Cplane2	-3.81	4.20E-02	1.00E+00
Dkkl1	-3.79	2.20E-02	1.00E+00
Kcnip4	-3.65	3.43E-02	1.00E+00
F630028O10Rik	-3.64	2.14E-02	1.00E+00
Gm4951	-3.62	2.58E-02	1.00E+00
Afap1l2	-3.60	1.75E-03	8.23E-01
Cldn5	-3.56	2.86E-02	1.00E+00
F5	-3.56	4.22E-02	1.00E+00
AA465934	-3.55	3.00E-02	1.00E+00
Tmem200b	-3.53	3.50E-02	1.00E+00
Lrrn2	-3.53	3.67E-02	1.00E+00
B230319C09Rik	-3.52	2.65E-02	1.00E+00
Pde10a	-3.40	8.59E-03	1.00E+00
Insyn2a	-3.36	3.45E-02	1.00E+00
Trank1	-3.31	1.15E-02	1.00E+00
Rad54l	-3.27	1.92E-02	1.00E+00
Gpr22	-3.27	1.41E-02	1.00E+00
Ankrd9	-3.24	2.29E-02	1.00E+00
Il1rl1	-3.18	3.54E-02	1.00E+00
Zcchc18	-3.17	3.41E-02	1.00E+00
Slc4a1	-3.15	3.60E-02	1.00E+00

Crp	-3.14	4.87E-03	1.00E+00
Fbxw10	-3.11	4.51E-02	1.00E+00
Gm336	-3.07	3.33E-02	1.00E+00
Sema3d	-2.96	1.12E-03	6.60E-01
Prtg	-2.90	3.47E-02	1.00E+00
Zfp354b	-2.87	3.83E-02	1.00E+00
Cfap300	-2.86	1.90E-02	1.00E+00
Cdh13	-2.82	1.05E-02	1.00E+00
Rinl	-2.79	3.10E-02	1.00E+00
Gm16023	-2.78	3.46E-02	1.00E+00
Slc4a8	-2.76	3.95E-02	1.00E+00
Itga8	-2.76	4.35E-02	1.00E+00
Igf2	-2.70	4.99E-02	1.00E+00
4930565N06Rik	-2.65	1.91E-02	1.00E+00
Lvrn	-2.56	4.29E-03	1.00E+00
Zfp128	-2.41	3.07E-02	1.00E+00
Snhg20	-2.37	2.03E-02	1.00E+00
Kcnh7	-2.35	4.10E-02	1.00E+00
Zfp185	-2.28	2.19E-02	1.00E+00
Abcg8	-2.26	2.65E-02	1.00E+00
Sgpp2	-2.21	2.88E-02	1.00E+00
Xaf1	-2.16	1.77E-02	1.00E+00
Sphkap	-2.16	3.97E-03	1.00E+00
Sh3tc2	-2.14	1.42E-02	1.00E+00
Cx3cr1	-2.13	2.72E-03	1.00E+00
Shld2	-2.11	1.28E-02	1.00E+00
Plcd3	-2.09	3.48E-02	1.00E+00
Ttn	-2.06	4.63E-03	1.00E+00
Zkscan16	-2.01	4.90E-02	1.00E+00
Lmln	-2.00	4.78E-02	1.00E+00
Efcab7	-2.00	4.53E-02	1.00E+00
B3galt2	-1.99	2.15E-02	1.00E+00
Gabbr1	-1.97	2.58E-02	1.00E+00
Wdhd1	-1.94	3.04E-02	1.00E+00
Tmem215	-1.92	4.69E-02	1.00E+00
Acy3	-1.85	2.68E-02	1.00E+00
Cacna2d1	-1.83	6.11E-03	1.00E+00
Plxna4	-1.82	2.88E-03	1.00E+00
Slc2a2	-1.75	2.11E-02	1.00E+00
Snapc3	-1.74	2.66E-02	1.00E+00
Ccdc126	-1.73	3.83E-02	1.00E+00
Alas2	-1.67	9.45E-03	1.00E+00
Cwc27	-1.65	2.73E-02	1.00E+00
Nsun6	-1.64	2.70E-02	1.00E+00
Chaserr	-1.63	3.23E-02	1.00E+00

Meg3	-1.62	3.30E-02	1.00E+00
Zfp945	-1.62	5.09E-03	1.00E+00
Nhsl2	-1.58	2.47E-02	1.00E+00
Acp1	-1.58	1.63E-03	8.23E-01
Zbtb34	-1.55	1.06E-03	6.43E-01
Hba-a1	-1.54	1.74E-03	8.23E-01
Tmem229a	-1.52	4.46E-03	1.00E+00
Nexmif	-1.51	1.92E-02	1.00E+00
Cep290	-1.51	9.62E-03	1.00E+00
Rspo1	-1.49	4.46E-02	1.00E+00
Zfp668	-1.48	4.35E-02	1.00E+00
Lrif1	-1.42	1.12E-02	1.00E+00
Syt7	-1.42	3.48E-02	1.00E+00
Tmod2	-1.37	1.38E-02	1.00E+00
Aasdh	-1.37	3.47E-02	1.00E+00
Akt3	-1.36	1.67E-02	1.00E+00
Slc4a4	-1.33	1.80E-02	1.00E+00
Btnl9	-1.33	4.42E-02	1.00E+00
Cep170	-1.32	2.34E-02	1.00E+00
Lox	-1.31	1.80E-02	1.00E+00
Hba-a2	-1.29	2.11E-02	1.00E+00
Cyyr1	-1.29	8.99E-03	1.00E+00
Dbn1	-1.29	2.95E-02	1.00E+00
Pear1	-1.28	3.49E-02	1.00E+00
Rbms3	-1.27	5.80E-03	1.00E+00
Sema6a	-1.26	2.48E-02	1.00E+00
Pld1	-1.26	3.27E-03	1.00E+00
Mtss2	-1.25	4.29E-02	1.00E+00
Cep41	-1.24	4.90E-02	1.00E+00
Zfp595	-1.23	3.41E-02	1.00E+00
Rnpc3	-1.23	1.95E-02	1.00E+00
Pdgfd	-1.21	2.69E-02	1.00E+00
Per2	-1.20	1.10E-02	1.00E+00
Parp14	-1.19	2.44E-02	1.00E+00
Mir1938	-1.19	2.90E-02	1.00E+00
4632427E13Rik	-1.18	2.32E-02	1.00E+00
Pigr	-1.17	1.25E-03	7.02E-01
Dcdc2a	-1.16	4.42E-02	1.00E+00
Zbtb20	-1.14	4.16E-04	2.99E-01
Acaca	-1.14	2.69E-02	1.00E+00
Pdzd2	-1.13	3.61E-02	1.00E+00
Notch4	-1.13	3.56E-02	1.00E+00
Cramp1	-1.13	6.88E-03	1.00E+00
Slc43a3	-1.13	1.56E-03	8.23E-01
Malat1	-1.12	1.47E-04	1.21E-01

Cntrl	-1.11	2.13E-02	1.00E+00
Hmcn1	-1.11	4.61E-02	1.00E+00
Peg3	-1.11	8.16E-03	1.00E+00
Adgrg6	-1.11	3.87E-02	1.00E+00
Zfp322a	-1.10	1.70E-02	1.00E+00
Scg2	-1.09	1.02E-02	1.00E+00
Zfp704	-1.09	2.77E-03	1.00E+00
Pds5b	-1.08	2.87E-03	1.00E+00
Gemin6	-1.08	3.06E-02	1.00E+00
Hook3	-1.08	3.53E-03	1.00E+00
Ylpm1	-1.08	1.34E-02	1.00E+00
Ganc	-1.07	2.86E-02	1.00E+00
Cobl	-1.07	1.24E-02	1.00E+00
Kctd12	-1.07	8.91E-03	1.00E+00
Mme	-1.06	4.80E-02	1.00E+00
Eif2ak2	-1.05	1.99E-02	1.00E+00
Irak4	-1.05	4.47E-02	1.00E+00
Slc40a1	-1.05	2.50E-03	1.00E+00
Myof	-1.04	1.98E-02	1.00E+00
Zbtb21	-1.04	9.99E-03	1.00E+00
Limch1	-1.02	4.79E-02	1.00E+00
Atp1b1	-1.00	1.90E-02	1.00E+00
Cops9	1.01	3.50E-03	1.00E+00
U2af1l4	1.03	4.62E-02	1.00E+00
Lcat	1.04	1.72E-03	8.23E-01
Gm3571	1.04	4.91E-02	1.00E+00
Lyplal1	1.05	8.48E-03	1.00E+00
Tspan1	1.06	1.74E-02	1.00E+00
H2az1	1.06	2.63E-03	1.00E+00
Nsdhl	1.06	1.97E-02	1.00E+00
E2f1	1.08	4.83E-02	1.00E+00
Mrps21	1.08	5.00E-02	1.00E+00
Csrnp1	1.09	1.80E-02	1.00E+00
Arhgap45	1.23	2.37E-02	1.00E+00
Dusp23	1.24	3.20E-02	1.00E+00
Prss1	1.29	9.47E-04	5.90E-01
Tnfaip3	1.31	1.08E-02	1.00E+00
Nr4a1	1.32	2.12E-03	9.78E-01
Thy1	1.34	2.13E-02	1.00E+00
H2bc13	1.34	4.00E-02	1.00E+00
H4c12	1.38	3.53E-02	1.00E+00
Fgd1	1.43	2.02E-02	1.00E+00
Fdps	1.47	2.04E-05	1.81E-02
Gm5771	1.49	3.32E-06	5.10E-03
Lrrc73	1.57	4.41E-02	1.00E+00

Prxl2b	1.60	1.09E-02	1.00E+00
Ttc39c	1.67	4.07E-02	1.00E+00
Prdm5	1.70	2.78E-02	1.00E+00
Shisa4	1.71	2.01E-02	1.00E+00
H1f5	1.72	3.78E-02	1.00E+00
Egr2	1.78	8.62E-03	1.00E+00
Atf3	1.79	1.86E-06	3.06E-03
Cfap43	1.87	4.86E-02	1.00E+00
Psd2	1.89	4.71E-02	1.00E+00
Dkk3	1.93	3.82E-02	1.00E+00
H4c6	1.95	5.48E-03	1.00E+00
Tspan11	1.97	4.38E-02	1.00E+00
Gm24044	2.05	3.59E-02	1.00E+00
5830444B04Rik	2.13	3.61E-02	1.00E+00
Gm22579	2.25	1.89E-02	1.00E+00
Milr1	2.30	2.79E-02	1.00E+00
Rtn2	2.35	1.90E-02	1.00E+00
Map4k1	2.37	1.73E-02	1.00E+00
Cd83	2.41	2.60E-04	2.06E-01
Rgs10	2.46	1.78E-02	1.00E+00
Dusp2	2.51	3.86E-02	1.00E+00
Gm10069	2.51	4.15E-02	1.00E+00
Hexim2	2.66	1.89E-02	1.00E+00
Gm8425	2.72	1.90E-02	1.00E+00
5033430I15Rik	3.21	1.90E-02	1.00E+00
Trbc2	3.21	1.88E-02	1.00E+00
Ighg3	3.26	4.76E-02	1.00E+00
Edil3	3.28	2.64E-02	1.00E+00
Ptprr	3.38	3.25E-02	1.00E+00
Cd69	3.40	2.76E-02	1.00E+00
Zmynd12	3.43	1.62E-02	1.00E+00
Gm8369	3.48	4.34E-02	1.00E+00
Trarg1	3.57	1.24E-02	1.00E+00
Esrrg	3.63	4.22E-05	3.61E-02
Fcmr	3.66	4.88E-02	1.00E+00
Pclaf	3.76	3.29E-02	1.00E+00
Ccr7	3.81	2.90E-02	1.00E+00
Lmo1	3.99	3.03E-02	1.00E+00
Was	4.01	4.63E-03	1.00E+00
Ccdc24	4.04	2.73E-02	1.00E+00
C920021L13Rik	4.11	2.60E-02	1.00E+00
Ccnb1	4.27	4.29E-02	1.00E+00
Fkbp1b	4.30	1.98E-02	1.00E+00
Rasd2	4.31	2.42E-02	1.00E+00
Grap2	4.44	8.50E-03	1.00E+00

Slco5a1	4.46	4.05E-02	1.00E+00
Car3	4.54	1.30E-02	1.00E+00
Rgs16	4.64	7.07E-03	1.00E+00
Chst3	4.90	7.74E-03	1.00E+00
Xcr1	4.94	9.82E-03	1.00E+00
Vpreb3	5.05	1.26E-02	1.00E+00
Igkv6-15	5.23	8.18E-03	1.00E+00
Igkv8-19	5.64	2.71E-02	1.00E+00
Nsg2	5.91	3.38E-03	1.00E+00
Xist	9.49	5.51E-03	1.00E+00
Cfd	10.90	4.70E-03	1.00E+00
Ighv1-77	19.48	1.87E-07	3.31E-04
Fam169b	19.49	1.16E-07	2.22E-04
Serpina3c	19.59	2.50E-08	6.41E-05
Slc36a2	19.92	8.01E-09	2.64E-05
Fcrl1	20.25	3.16E-11	1.46E-07
Retn	20.29	2.01E-11	1.16E-07
Acod1	20.45	5.46E-12	4.20E-08
Ighg2b	21.64	3.79E-08	8.74E-05

* expression in *Ptf1a* $^{cre/+}$ *KRAS* ^{G12D} relative to *APK*
