# Supplementary material for: Acinar-specific loss of activating transcription factor 3 restricts KRASG12D mediated transcriptional changes and PanIN progression
Source: Cell Death Discov. 2025 Nov 6;11:503. doi: 10.1038/s41420-025-02777-2 (PMC12592554; doi:10.1038/s41420-025-02777-2)
Supplement: Supplementary file 10 — Suplementary Table S3. DEGs between Ptf1a+/creERTKRASG12D and APK Organoids [file 41420_2025_2777_MOESM10_ESM.pdf]

Suplementary Table S3. DEGs between Ptf1a$^{+/creERT}$KRAS^{G12D} and APK Organoids

Symbol	Entrez Accession #	log2 Fold Change	p value	p adj
Gkn1	66283	-23.02	6.78E-08	3.79E-05
Galnt18	233733	-8.96	1.09E-08	9.30E-06
Cd300lb	217304	-6.46	9.25E-07	3.61E-04
Fndc1	68655	-6.05	2.78E-04	2.80E-02
P2rx7	18439	-5.47	1.31E-08	1.02E-05
Raet1e	379043	-5.37	6.98E-05	1.06E-02
Fst	14313	-5.34	3.01E-07	1.34E-04
Col4a1	12826	-4.95	4.64E-04	3.99E-02
Ramp3	56089	-4.76	1.81E-07	9.10E-05
E030030I06Rik	319887	-4.66	5.51E-04	4.41E-02
Clca3b	229927	-4.37	7.78E-09	7.60E-06
Pkp1	18772	-4.31	1.95E-06	6.26E-04
Zfp992	433791	-4.29	6.01E-04	4.61E-02
Dmbt1	12945	-4.27	9.71E-06	2.29E-03
Cidea	12683	-4.19	1.66E-05	3.52E-03
Gulp1	70676	-4.01	2.44E-04	2.53E-02
Trp53cor1	100504267	-3.81	2.10E-05	4.22E-03
Sorcs2	81840	-3.67	1.39E-04	1.67E-02
Lgals1	16852	-3.60	5.29E-04	4.35E-02
Zfp268	433801	-3.48	1.21E-04	1.50E-02
Wincr1	100040617	-3.47	5.52E-04	4.41E-02
Slc13a2	20500	-3.47	4.25E-04	3.75E-02
Megf6	230971	-3.35	4.60E-38	8.99E-34
Hebp2	56016	-3.31	2.80E-04	2.80E-02
Sult4a1	29859	-3.29	1.42E-04	1.69E-02
Htra1	56213	-3.19	4.12E-04	3.69E-02
Vav3	57257	-3.07	2.92E-07	1.33E-04
Trib2	217410	-3.07	7.04E-05	1.06E-02
Nupr1	56312	-3.07	2.86E-04	2.82E-02
Ptpn22	19260	-2.95	4.12E-04	3.69E-02
Trim47	217333	-2.88	2.92E-04	2.85E-02
St8sia3	20451	-2.79	2.61E-04	2.69E-02
Lhx9	16876	-2.78	5.71E-04	4.44E-02
Zmynd15	574428	-2.73	4.01E-08	2.62E-05
Gm5850	545543	-2.65	1.53E-04	1.79E-02
Srgap1	117600	-2.63	4.78E-04	4.06E-02
Lhfp	108927	-2.60	3.29E-04	3.07E-02
Tmem238l	71576	-2.58	2.07E-04	2.25E-02
Wnt7a	22421	-2.55	6.53E-04	4.85E-02
Nod2	257632	-2.54	8.32E-07	3.36E-04
Kctd14	233529	-2.45	1.03E-04	1.37E-02
Sema7a	20361	-2.38	6.69E-05	1.02E-02

Prss27	213171	-2.31	5.53E-04	4.41E-02
C1qtnf1	56745	-2.23	1.34E-04	1.62E-02
Pcsk9	100102	-2.16	1.60E-04	1.83E-02
Prex2	109294	-2.13	3.09E-05	5.59E-03
Atf3	11910	-2.13	6.54E-06	1.70E-03
Eomes	13813	-2.11	8.39E-05	1.20E-02
Mir31	723895	-2.11	3.92E-05	6.66E-03
Nipal2	223473	-2.11	1.73E-04	1.95E-02
Aif1l	108897	-2.10	2.02E-04	2.20E-02
Tigd4	403175	-2.09	5.01E-04	4.22E-02
Zdhhc15	108672	-2.06	1.82E-07	9.10E-05
Ankrd44	329154	-2.04	6.21E-04	4.70E-02
Gdpd5	233552	-2.00	3.22E-04	3.04E-02
Ccdc28b	66264	-1.99	3.23E-05	5.73E-03
Arid5a	214855	-1.98	4.26E-04	3.75E-02
Col7a1	12836	-1.89	6.17E-04	4.69E-02
Samd4	74480	-1.85	2.20E-05	4.26E-03
P4ha2	18452	-1.79	1.92E-05	3.96E-03
1700025G04Rik	69399	-1.76	2.77E-05	5.18E-03
Pear1	73182	-1.72	4.30E-04	3.77E-02
Tnip3	414084	-1.71	8.42E-07	3.36E-04
Zfp57	22715	-1.71	6.37E-04	4.75E-02
Pdzrn3	55983	-1.71	1.03E-05	2.37E-03
Mpdz	17475	-1.71	2.71E-06	8.27E-04
Pcdhgb6	93703	-1.61	2.19E-05	4.26E-03
Flnb	286940	-1.56	5.50E-06	1.47E-03
Slc25a45	107375	-1.55	5.78E-04	4.48E-02
Sh3pxd2a	14218	-1.51	4.42E-06	1.21E-03
Elapor1	229722	-1.44	3.84E-05	6.61E-03
Aplp1	11803	-1.40	1.54E-06	5.46E-04
Klf4	16600	-1.39	1.01E-04	1.35E-02
Asap3	230837	-1.39	2.16E-05	4.26E-03
Lurap1	68075	-1.36	1.64E-09	2.14E-06
Gm11714	108167930	-1.36	3.74E-04	3.46E-02
Clic4	29876	-1.31	4.12E-04	3.69E-02
Ttc39b	69863	-1.30	4.60E-05	7.42E-03
Lhfpl2	218454	-1.27	9.17E-05	1.28E-02
Itga3	16400	-1.27	3.90E-04	3.59E-02
Pik3r3	18710	-1.26	2.13E-04	2.27E-02
Btg2	12227	-1.24	7.75E-06	1.94E-03
Ogdhl	239017	-1.21	1.86E-04	2.04E-02
Rhoq	104215	-1.20	1.74E-06	5.77E-04
Ptpn13	19249	-1.17	5.83E-04	4.51E-02
Macf1	11426	-1.15	1.03E-05	2.37E-03
Parp16	214424	-1.13	1.61E-05	3.46E-03

Megf11	214058	-1.11	2.28E-04	2.40E-02
Insyn2a	627214	-1.10	4.52E-04	3.91E-02
Zc3h12a	230738	-1.10	1.41E-04	1.69E-02
Fosl2	14284	-1.09	1.12E-04	1.43E-02
Iffo2	212632	-1.08	4.95E-04	4.19E-02
Ccdc171	320226	-1.05	4.72E-04	4.02E-02
Dmxl2	235380	-1.05	9.30E-06	2.22E-03
Dusp10	63953	-1.04	6.14E-04	4.68E-02
Entpd2	12496	1.00	2.88E-04	2.82E-02
Idh1	15926	1.21	1.33E-04	1.62E-02
Blvrb	233016	1.21	5.55E-04	4.41E-02
Sdr42e1	74032	1.22	2.15E-05	4.26E-03
Gm12918	100039656	1.22	1.00E-04	1.35E-02
Klhl23	277396	1.24	7.58E-05	1.11E-02
H3c7	260423	1.24	3.13E-04	3.00E-02
Akr1c19	432720	1.25	7.76E-05	1.13E-02
Mpst	246221	1.27	7.76E-06	1.94E-03
Pla2g12a	66350	1.34	3.64E-04	3.39E-02
Pcolce	18542	1.37	7.38E-05	1.10E-02
Syce2	71846	1.37	3.13E-06	9.40E-04
H3c4	319149	1.42	2.08E-04	2.25E-02
H2ac20	319176	1.42	4.36E-04	3.78E-02
Gstt1	14871	1.44	1.69E-06	5.69E-04
Rab27b	80718	1.45	4.09E-09	4.99E-06
H2ac10	319173	1.46	2.13E-04	2.27E-02
H3c3	319148	1.50	4.14E-04	3.69E-02
H3c6	319151	1.52	5.53E-08	3.28E-05
H3c2	319150	1.55	4.37E-05	7.28E-03
H2ac22	319170	1.55	2.85E-04	2.82E-02
1810010H24Rik	69066	1.59	9.50E-05	1.32E-02
Ifi27l2b	217845	1.59	3.16E-04	3.01E-02
Foxa3	15377	1.64	6.49E-08	3.73E-05
Tst	22117	1.66	2.50E-05	4.74E-03
Psat1	107272	1.67	1.04E-04	1.37E-02
Gask1b	68659	1.71	5.03E-04	4.22E-02
Dennd5b	320560	1.72	8.49E-05	1.20E-02
Fzd4	14366	1.73	3.15E-05	5.64E-03
H3c8	97908	1.73	1.52E-04	1.79E-02
H3c1	360198	1.74	1.15E-04	1.45E-02
H4c16	320332	1.75	6.93E-09	7.12E-06
Gjb1	14618	1.80	4.41E-06	1.21E-03
Dnm3	103967	1.80	5.33E-08	3.26E-05
Nkain4	58237	1.94	3.86E-05	6.61E-03
Radx	102871	1.95	1.56E-04	1.80E-02
Slc39a8	67547	1.98	2.86E-07	1.33E-04

Ccdc154	207209	2.00	3.08E-07	1.34E-04
Fetub	59083	2.03	2.15E-08	1.55E-05
Lzts3	241638	2.04	1.72E-07	9.10E-05
Arhgap24	231532	2.06	5.70E-04	4.44E-02
Tspan12	269831	2.07	7.03E-06	1.81E-03
Gpam	14732	2.13	1.01E-04	1.35E-02
Ptar1	72351	2.15	4.30E-13	1.20E-09
C1rl	232371	2.24	1.20E-08	9.76E-06
Pcdhga7	93715	2.30	1.21E-09	1.71E-06
C1ra	50909	2.44	2.67E-04	2.73E-02
Gstm1	14862	2.48	1.45E-04	1.72E-02
Entrep1	381217	2.52	7.78E-05	1.13E-02
Ly6g2	223631	2.56	1.88E-05	3.90E-03
Cfi	12630	2.60	5.65E-04	4.43E-02
Ros1	19886	2.68	1.08E-08	9.30E-06
Clic5	224796	2.68	9.30E-06	2.22E-03
Ugt2b35	243085	2.69	1.10E-12	2.38E-09
Abca8b	27404	2.69	6.36E-04	4.75E-02
Aldh1a7	26358	2.77	3.94E-08	2.62E-05
Adh7	11529	2.80	2.23E-05	4.27E-03
Itih2	16425	2.84	1.35E-05	2.96E-03
Amdhd1	71761	2.92	3.01E-04	2.91E-02
Chpt1	212862	2.93	1.51E-13	4.92E-10
Gstm2	14863	3.10	5.38E-05	8.35E-03
Ighv6-4	628398	3.13	4.39E-06	1.21E-03
Fras1	231470	3.18	2.32E-04	2.43E-02
Bche	12038	3.25	1.69E-06	5.69E-04
Adora1	11539	3.27	2.68E-07	1.28E-04
Kcnk2	16526	3.32	6.41E-05	9.85E-03
Fgf9	14180	3.36	4.40E-05	7.28E-03
Hp	15439	3.39	1.35E-33	1.32E-29
Gpr35	64095	3.45	7.63E-13	1.86E-09
Id3	15903	3.48	5.87E-07	2.44E-04
Fgfr3	14184	3.49	8.11E-19	3.17E-15
Bhlhb9	70237	3.55	9.01E-05	1.27E-02
Gstm3	14864	3.56	6.56E-04	4.86E-02
Itga7	16404	3.57	8.72E-09	8.11E-06
Cdh16	12556	3.57	5.21E-07	2.21E-04
Rps23rg1	546049	3.58	1.68E-05	3.53E-03
Sytl2	83671	3.61	2.78E-05	5.18E-03
Cox6b2	333182	3.68	4.30E-05	7.23E-03
Klf15	66277	3.72	4.88E-05	7.75E-03
Gstt2	14872	3.74	3.29E-05	5.73E-03
Hif3a	53417	3.75	9.88E-08	5.36E-05
Nid2	18074	3.75	1.11E-23	5.40E-20

1700039E22Rik	73322	3.84	5.49E-04	4.41E-02
Aass	30956	3.90	2.15E-04	2.27E-02
Hcn1	15165	3.93	4.23E-06	1.21E-03
Tph2	216343	4.06	5.95E-04	4.58E-02
Bmp7	12162	4.12	1.30E-05	2.92E-03
Cyp2c65	72303	4.16	2.98E-05	5.49E-03
Afm	280662	4.82	2.34E-04	2.43E-02
Psg16	26436	5.01	5.60E-04	4.42E-02
Mettl7b	71664	5.05	1.05E-06	3.96E-04
Reln	19699	5.10	5.90E-09	6.40E-06
Slitrk6	239250	5.20	6.01E-06	1.59E-03
Slc17a2	218103	5.21	1.98E-05	4.03E-03
Apba1	319924	5.21	3.38E-06	1.00E-03
Serpina5	268591	5.69	1.08E-04	1.39E-02
Atp6v0a4	140494	5.93	3.43E-06	1.00E-03
Fign	60344	6.04	1.61E-08	1.21E-05
Cyp2c68	433247	6.41	9.21E-06	2.22E-03
Apoc4	11425	6.55	2.77E-04	2.80E-02
Zbtb16	235320	6.56	4.02E-08	2.62E-05
Ppp1r3c	53412	7.00	2.87E-12	5.10E-09
Ces1e	13897	7.01	3.27E-05	5.73E-03
Cndp1	338403	7.38	1.58E-04	1.82E-02
Ces1g	12623	7.73	2.05E-06	6.47E-04
Adh1	11522	7.84	1.13E-06	4.15E-04
Trim71	636931	8.93	1.69E-12	3.31E-09
Gm8909	667977	9.02	6.24E-10	1.02E-06
Apoc1	11812	10.60	3.24E-24	2.11E-20
Dlk1	13386	11.10	4.88E-09	5.60E-06
Upk3a	22270	17.73	2.11E-07	1.03E-04
Adh6a	69117	22.88	5.07E-08	3.20E-05
