# Supplementary material for: Acinar-specific loss of activating transcription factor 3 restricts KRASG12D mediated transcriptional changes and PanIN progression
Source: Cell Death Discov. 2025 Nov 6;11:503. doi: 10.1038/s41420-025-02777-2 (PMC12592554; doi:10.1038/s41420-025-02777-2)
Supplement: Supplementary file 11 — Supplementary figures [file 41420_2025_2777_MOESM11_ESM.pdf]

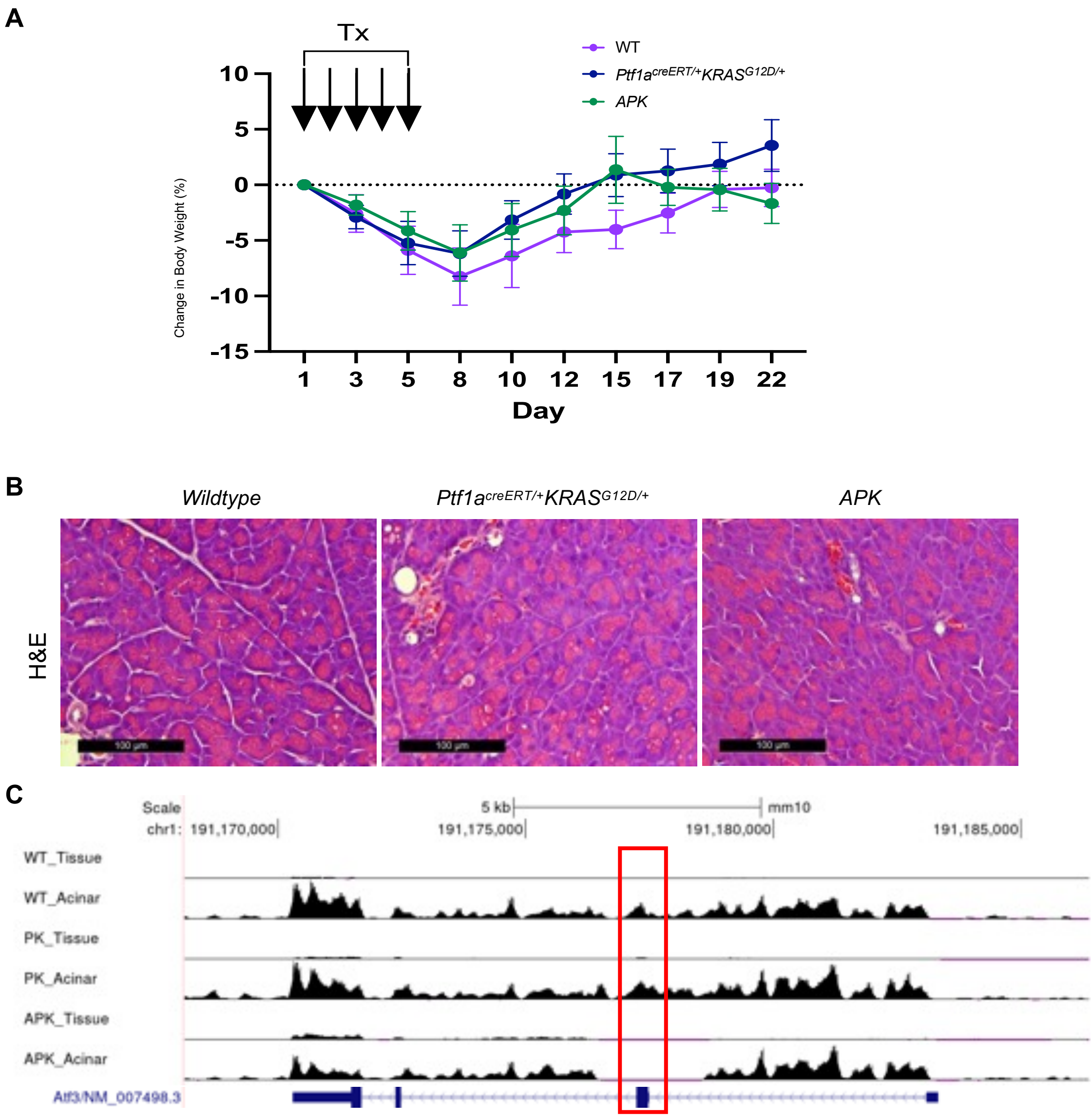

Supplemental Figure S1. Martin et al 2025. (A) Change in body weight following tamoxifen gavage (arrows). Data displayed as mean ± SEM. N = 9, 10, and 9 for WT, *Ptf1a*$^{creERT/+}$*KRAS*$^{G12D/+}$, and *APK,* mice respectively. **(B)** Representative H&E staining 22 days after after activating KRAS^{G12D} expression, *Ptf1a*$^{creERT/+}$*KRAS*$^{G12D/+}$ and *APK* mice show no differences in tissue morphology. Magnification bars = 200 µm. **(C)** *Atf3* gene tracks from RNA-seq analysis of *WT*, *Ptf1a*$^{creERT/+}$*KRAS*$^{G12D/+}$, and *APK* whole pancreatic tissue or isolated acinar cells showing deletion of exon 2 in *APK* mice and cells. Exon 2 carries the translational start site of *ATF3* mRNA.

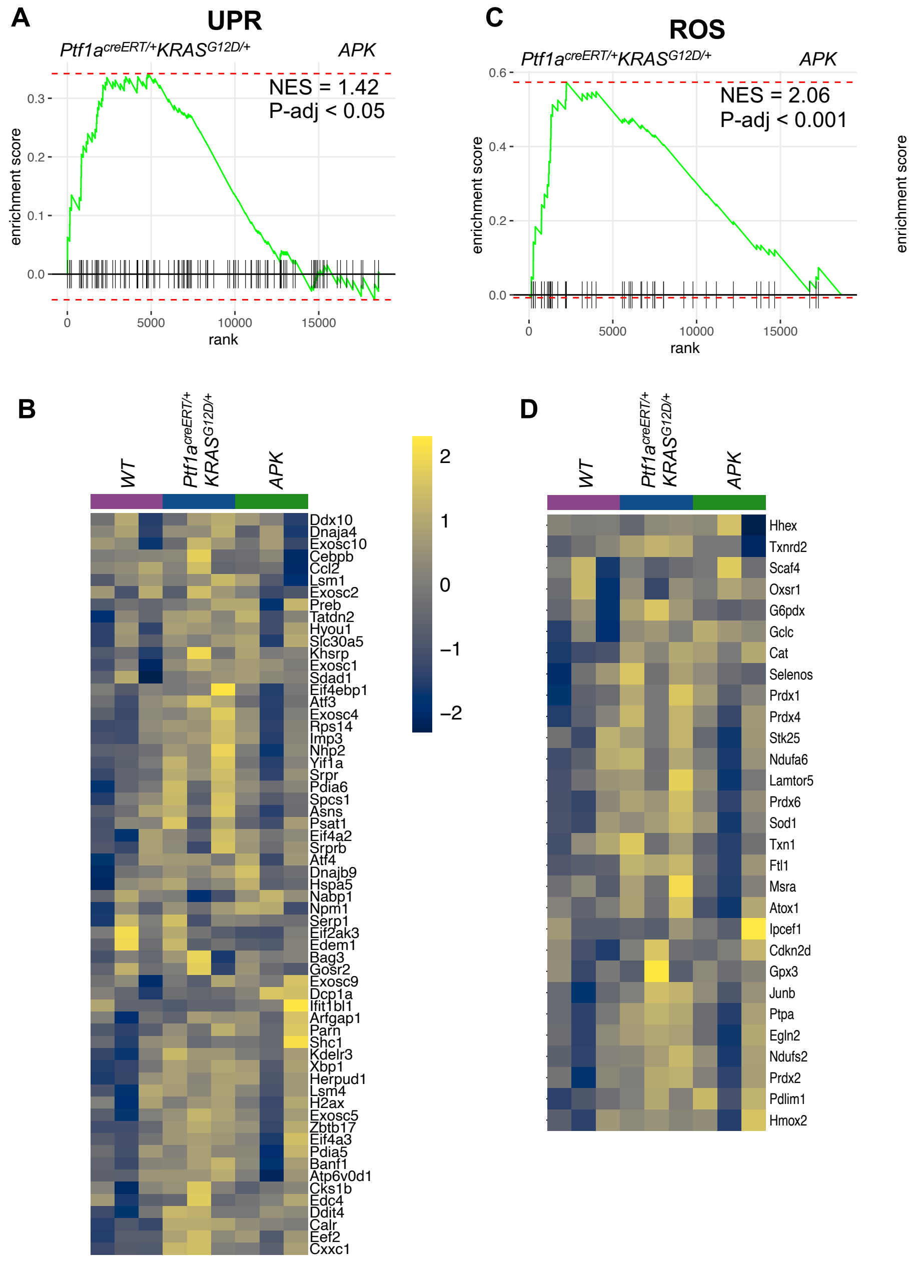

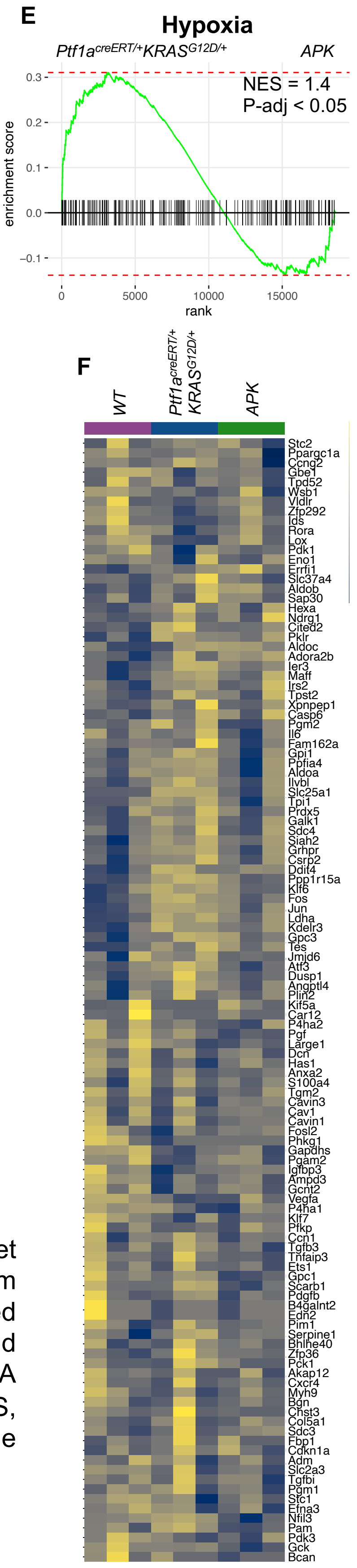

Supplemental Figure S2. Martin et al 2025. Gene Set Enrichment Analysis (GSEA) comparing RNA sequencing from *Ptf1a*$^{creERT/+}$*KRAS*$^{G12D/+}$ and *APK* acinar cells for **(A)** the unfolded protein response (UPR), **(C)** reactive oxygen species (ROS), and **(E)** hypoxia pathways. Heat maps depicting genes from GSEA hallmarks of **(B)** UPR, **(D)** ROS, and **(F)** hypoxia. NES, normalized enrichment score. Each lane represents a unique organoid line.

A **TNFα**

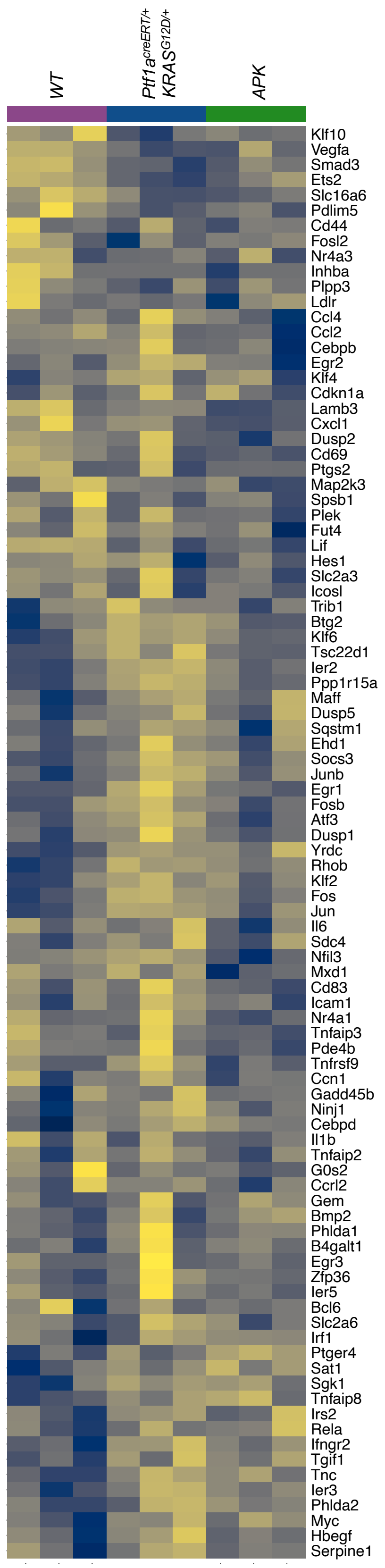

B **KRAS Signaling Up**

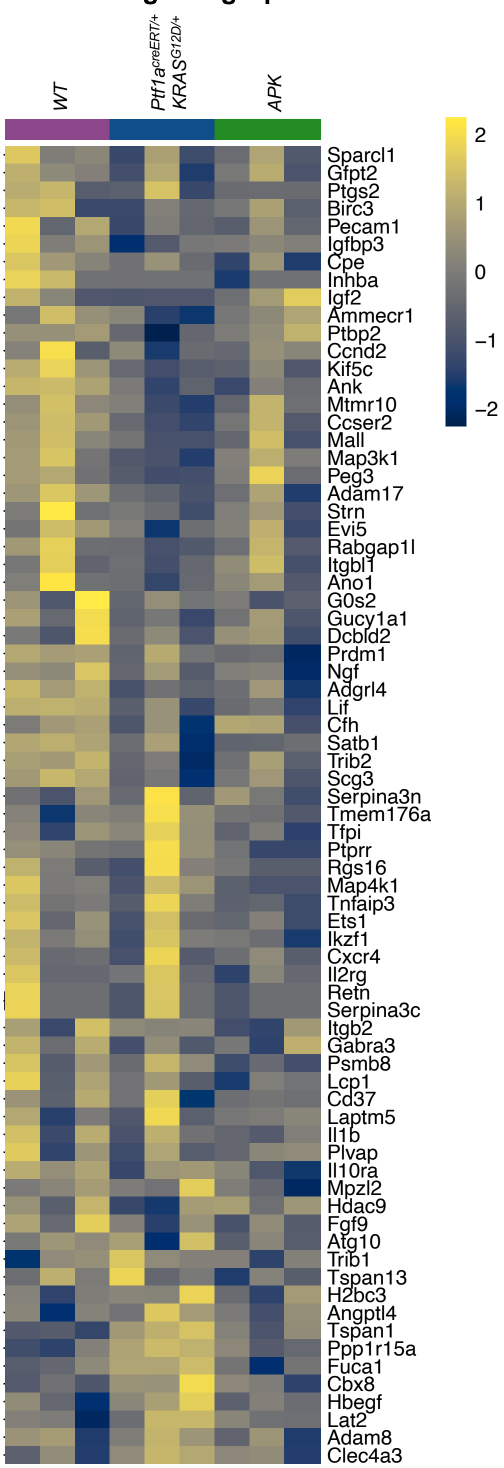

Supplemental Figure S3. Martin et al 2025. Heat maps from Gene Set Enrichment Analysis (GSEA) comparing RNA-seq from wild type, *Ptf1a*$^{creERT/+}$*KRAS*$^{G12D/+}$ and *APK* acinar cells for **(A)** TNFα and **(B)** KRAS Signaling Up pathways. NES, normalized enrichment score. Each lane represents a unique organoid line.

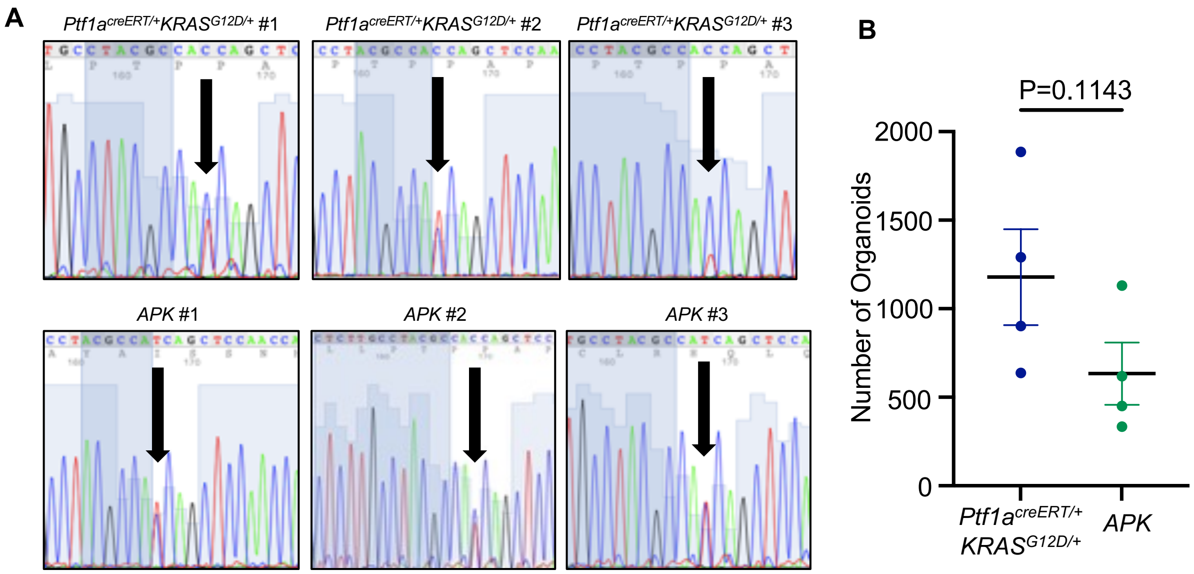

Supplemental Figure S4. Martin et al, 2025. Sequencing confirms acinar cell origin of organoids lines. (A) Sanger sequencing of cDNA amplifying KRAS in RNA isolated from $Ptf1a^{creERT/+}KRAS^{G12D/+}$ and *APK* organoid lines (n=3) confirmed heterozygous expression of $KRAS^{G12D}$ except $Ptf1a^{creERT/+}KRAS^{G12D/+}$ line #3 which showed higher levels of the $KRAS^{G12D}$ transcript. Black arrow indicates the position of the mutation corresponding for matching T and C nucleotide calls. (B) Quantification of number of organoids observed in $Ptf1a^{creERT/+}KRAS^{G12D/+}$ and *APK* cultures 7 days after passage displayed as mean ± SEM for n=4. Statistical analysis by t-test with p-value indicated.

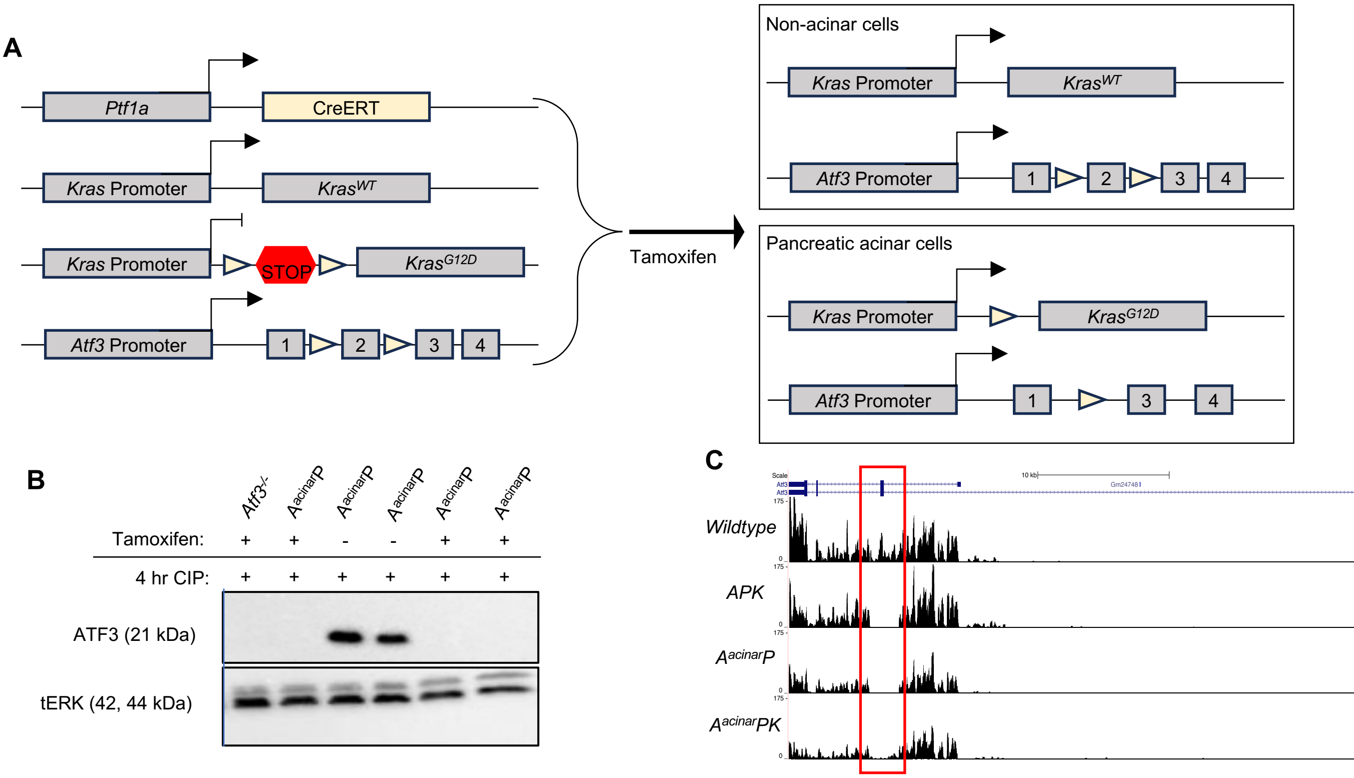

Supplemental Figure S5. Martin et al, 2025. (A) Schematic of acinar specific deletion of ATF3. **(B)** Western blot analysis for ATF3 on protein extracted from whole tissue of corn oil control or tamoxifen treated *A^acinar^P* animals 4 hours after cerulein treatment. Western blot shows deletion of ATF3 follow tamoxifen treatment. **(C)** *Atf3* gene tracks from based on RNA-seq from wild type, *APK*, $A^{acinar}P$ ($Atf3^{fl/fl}Ptf1a^{+/creERT}$) and $A^{acinar}PK$ acinar cells showing loss of exon 2.

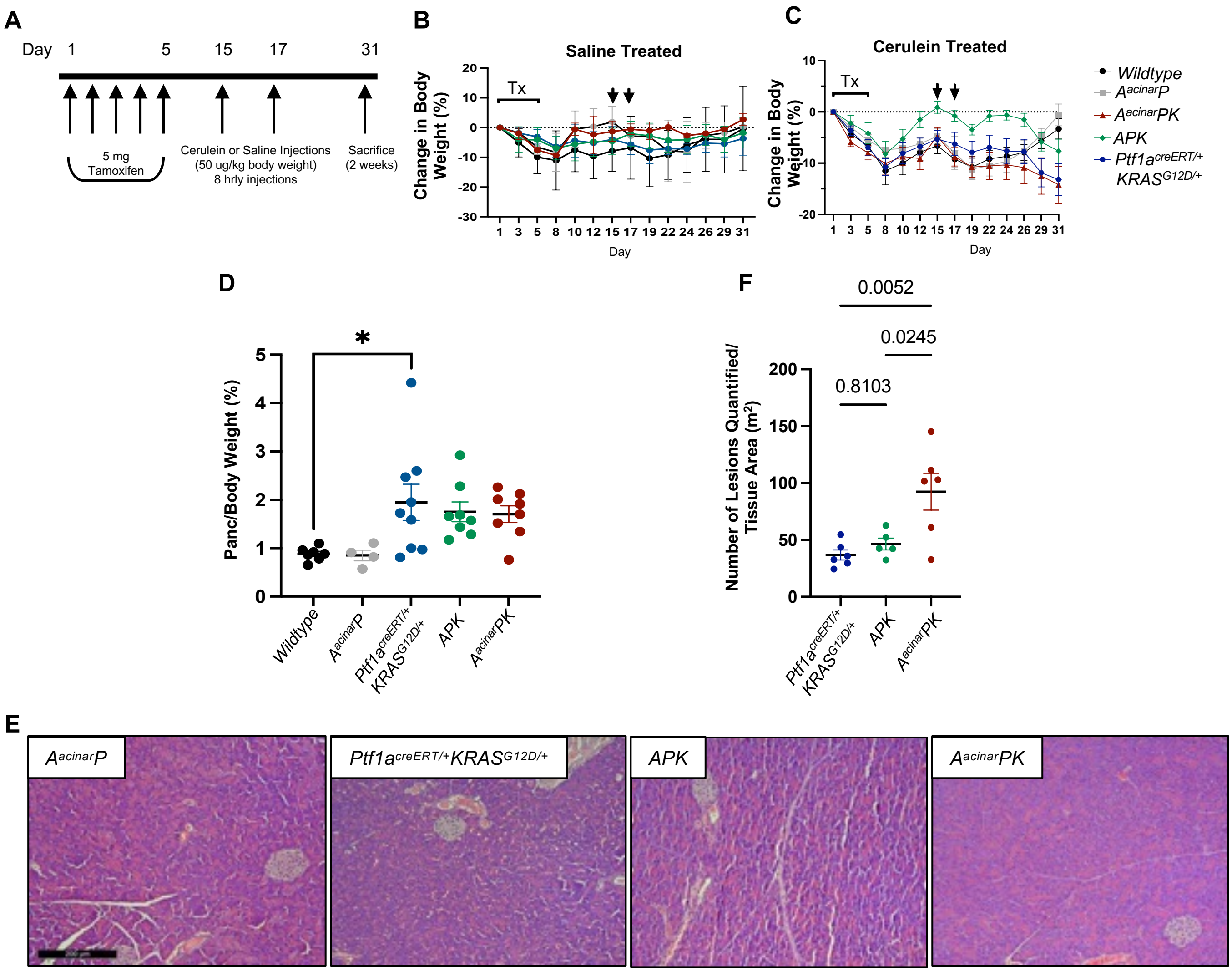

Supplemental Figure S6. Martin et al, 2025. (A) Schematic of 2-week cerulein treatment timeline. Change in body weight over experiment in **(B)** saline and **(C)** cerulein treated mice. Arrows indicate when cerulein was given. **(D)** Pancreatic weight as a ratio to body weight following cerulein treatment of wild type (n=7), $Ptf1a^{creERT/+}KRAS^{G12D/+}$ (n=9), *APK* (n=8), $A^{acinar}P$ ($Atf3f^{l/fl}Ptf1a^{+/creERT}$; n=4) and $A^{acinar}PK$ (n=8). Each dot represents an individual mouse. *P<0.05. **(E)** Representative H&E staining of saline-treated mice of indicated genotype. Magnification bars = 200 µm. **(F)** Number of quantified CK19+ lesions relative to tissue area in $Ptf1a^{creERT/+}KRAS^{G12D/+}$ (n=6), *APK* (n=5), and $A^{acinar}PK$ (n=6). . Data is displayed as mean ± SEM for panels **B-D, F**. Statistical analysis is one-way ANOVA with Tukey post-hoc with p-values indicated.

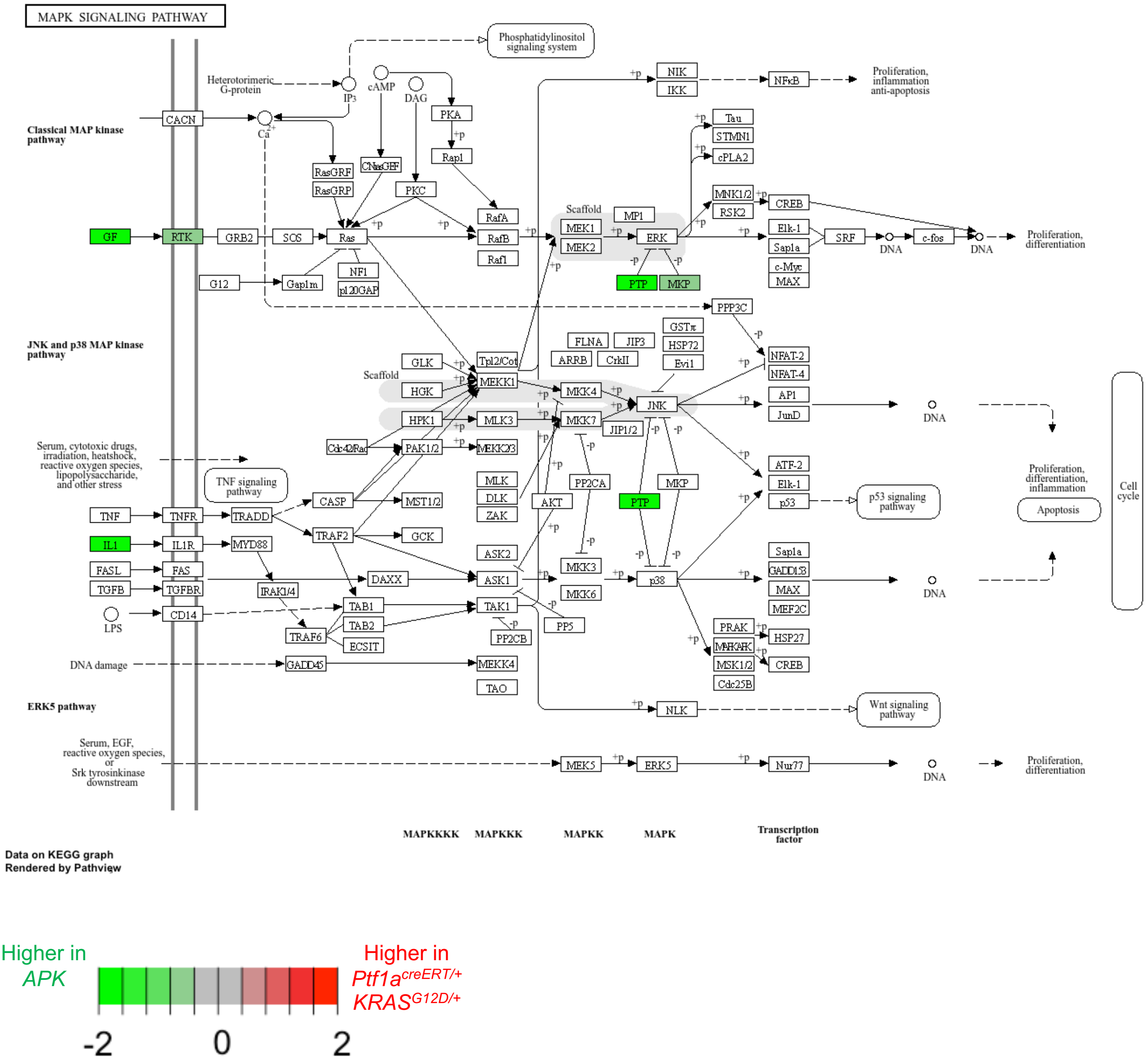

Supplemental Figure S7 Martin et al, 2025. KEGG Pathway Map for the MAPK Signaling pathway displaying gene expression of DEGs based on RNA-seq analysis between *Ptf1a*$^{creERT/+}$*KRAS*$^{G12D/+}$ and *APK* organoids (n=3 for each genotype). Genes in green are increased in *APK* organoids.
