# Supplementary material for: Acinar-specific loss of activating transcription factor 3 restricts KRASG12D mediated transcriptional changes and PanIN progression
Source: Cell Death Discov. 2025 Nov 6;11:503. doi: 10.1038/s41420-025-02777-2 (PMC12592554; doi:10.1038/s41420-025-02777-2)
Supplement: Supplementary file 12 — Complete Western blots [file 41420_2025_2777_MOESM12_ESM.pdf]

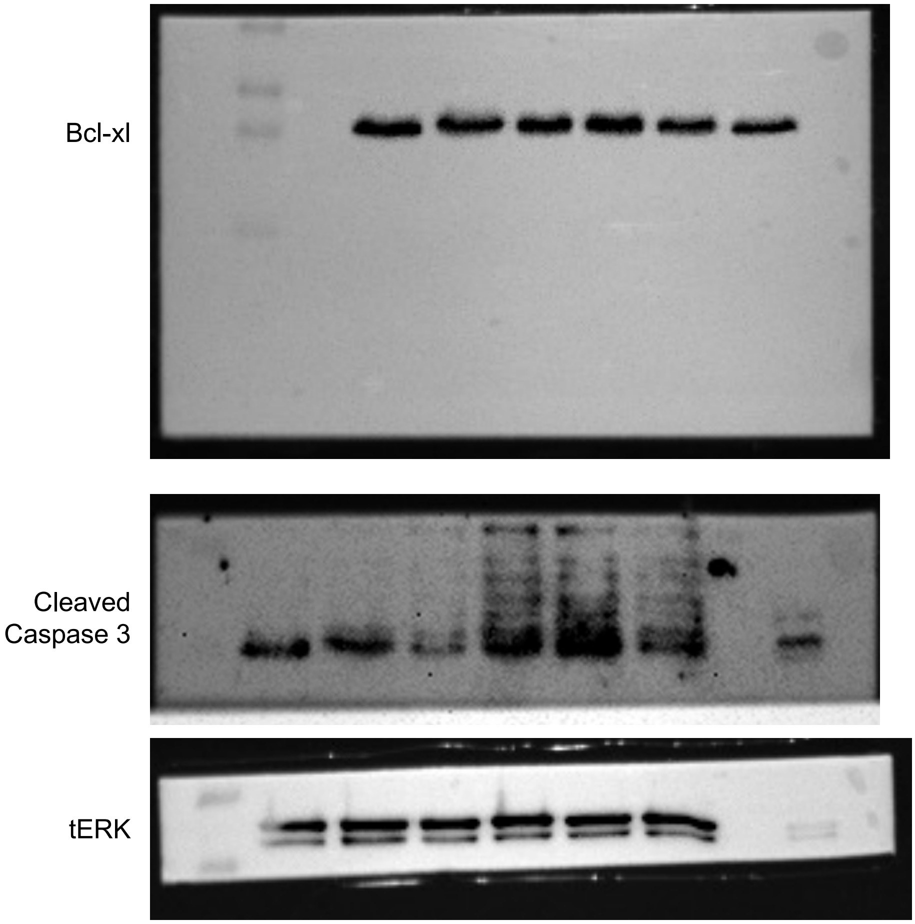

Supplemental Data. Full western blots for Figure 4E

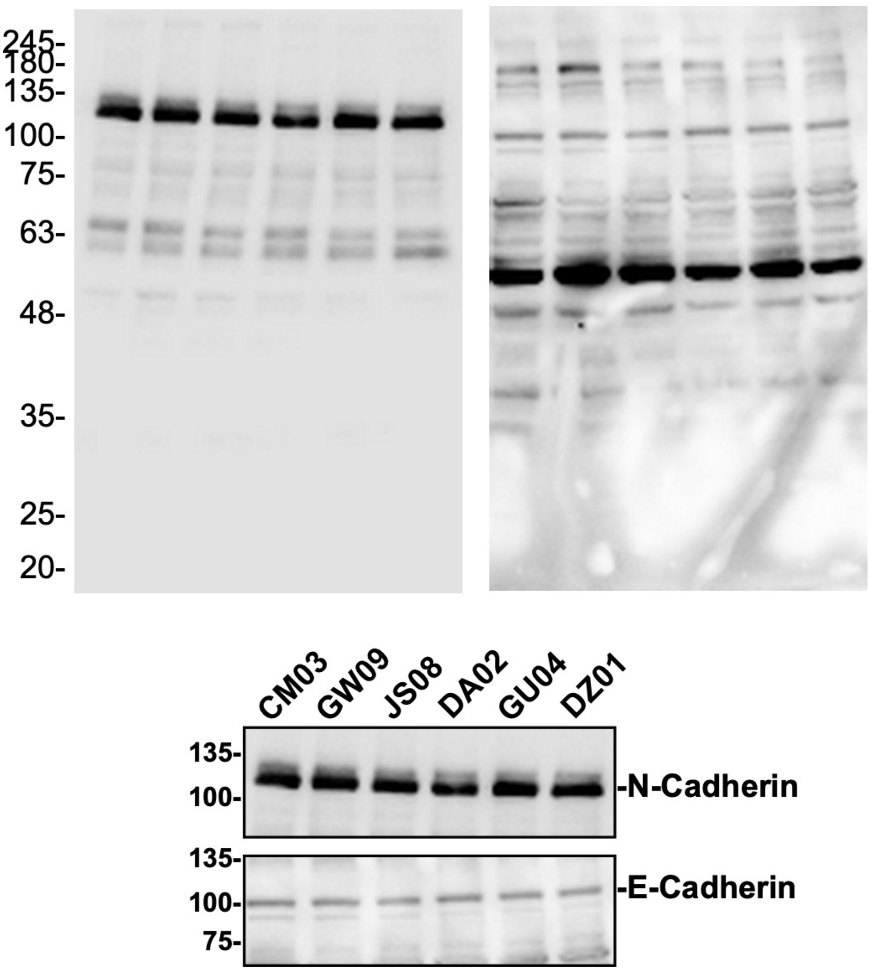

Supplemental Data. Full western blots for Figure 5G.

Total ERK

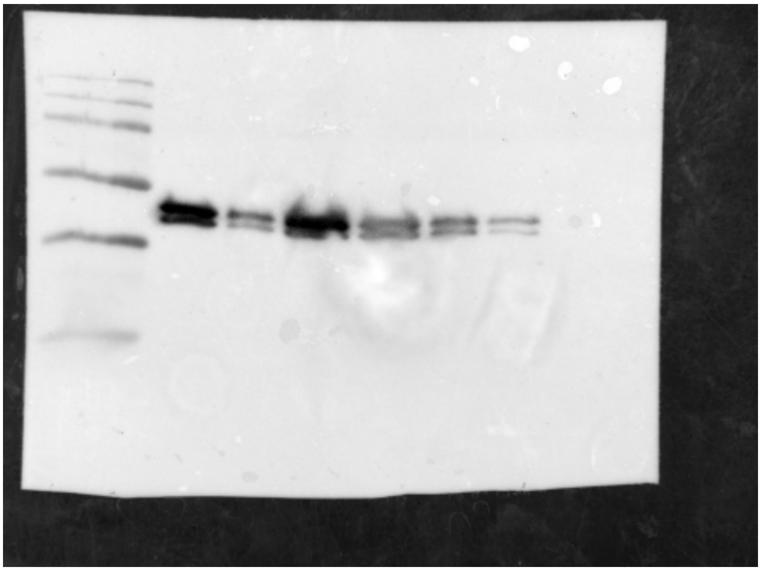

Phospho-ERK

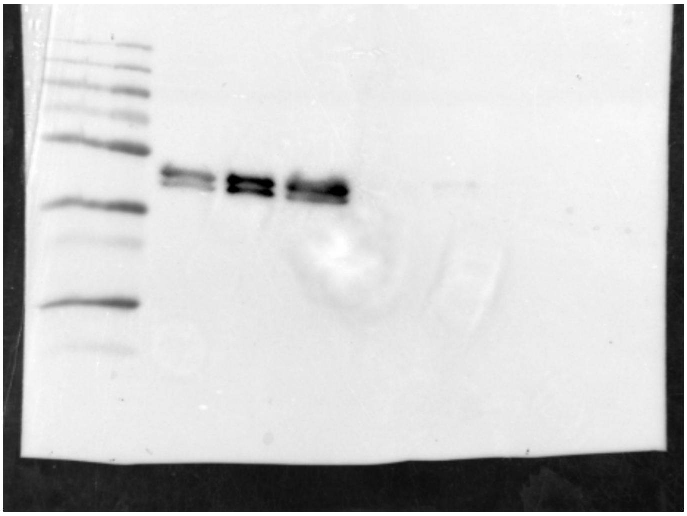

Supplemental Data. Full western blots for Figure 7G.

ATF3 (21 kDa)

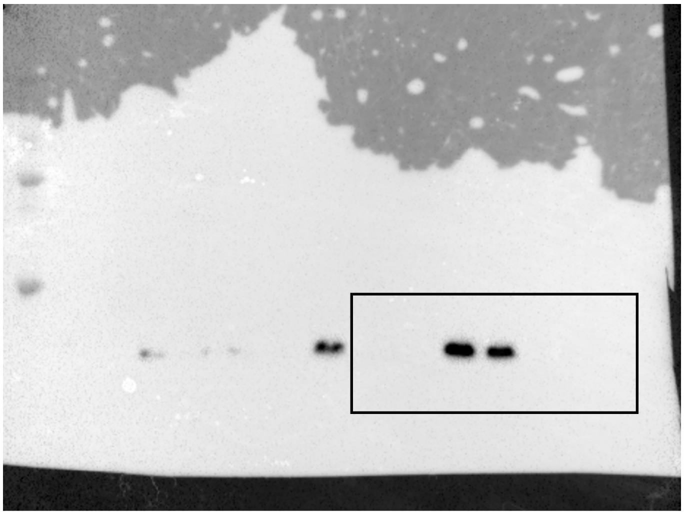

tERK (42, 44 kDa)

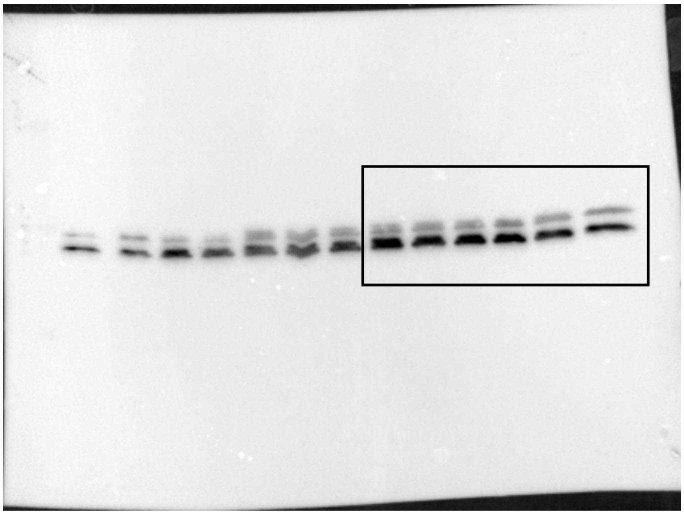

Supplemental Data. Full western blots for Supplemental Figure S3B. Lanes used for Figure are boxed.
